# Night eating in timing, frequency, and food quality and risks of all-cause, cancer, and diabetes mortality: findings from national health and nutrition examination survey

**DOI:** 10.1038/s41387-024-00266-6

**Published:** 2024-02-27

**Authors:** Peng Wang, Qilong Tan, Yaxuan Zhao, Jingwen Zhao, Yuzhu Zhang, Dan Shi

**Affiliations:** 1 Department of Nutrition and Food Hygiene, School of Public Health, Chongqing Medical University, Chongqing, China; 2 Department of Epidemiology and Biostatistics, School of Public Health, Zhejiang University School of Medicine, Hangzhou, Zhejiang China; 3 Research Centre for Environment and Human Health, School of Public Health, Chongqing Medical University, Chongqing, China; 4 Nutrition Innovation Platform-Sichuan and Chongqing, School of Public Health, Chongqing Medical University, Chongqing, China

**Keywords:** Nutrition, Lifestyle modification

## Abstract

**Objective:**

To investigate the association of timing, frequency, and food quality of night eating with all-cause, cancer, and diabetes mortality.

**Methods:**

This study included 41,744 participants from the US National Health and Nutrition Examination Survey (2002–2018). Night eating information was collected by 24-h dietary recall and the exposures were timing, frequency, and food quality of night eating. Food quality was assessed by latent class analysis. The outcomes were all-cause, cancer, and diabetes mortality, which were identified by the National Death Index and the International Classification of Diseases 10th Revision. Adjusted hazard ratios [aHR] with 95% confidence intervals [CI] were computed by Cox regression.

**Results:**

During a median follow-up of 8.7 years, 6066 deaths were documented, including 1381 from cancer and 206 from diabetes. Compared with no night eating (eating before 22:00), the later timing of night eating was associated with higher risk of all-cause and diabetes mortality (each *P*-trend <0.05) rather than cancer mortality, with the highest risk of eating being 00:00–1:00 (aHR 1.38, 95% CI 1.02–1.88) and being 23:00–00:00 (aHR 2.31, 95% CI 1.21–4.40), respectively. However, the increased risks were not observed for 22:00-23:00. Likewise, one time or over frequency of night eating was associated with higher all-cause and diabetes mortality (each *P* < 0.05). That risks were further observed in high-dietary-energy-density group of night eating (all-cause mortality: aHR 1.21 [95% CI 1.06–1.38]; diabetes mortality: aHR 1.97 [95% CI 1.13–3.45]), but not in low-dietary-energy-density group. Finally, correlation analysis found positive associations of night eating with glycohemoglobin, fasting glucose, and OGTT.

**Conclusions:**

Night eating was associated with increased all-cause, cancer and diabetes mortality; however, reduction of excess mortality risk was observed when eating before 23:00 or low-dietary-energy-density foods.

## Introduction

The time when meals are eaten is a significant indicator influencing overall health and well-being [1–3]. Night eating, characterized by the consumption of food during the late evening or nighttime hours, has been associated with various health risks, such as cancer [4, 5] and diabetes [6–8]. Despite intense investigation, the long-term health effect of night eating remains largely unclear. To our best acknowledge, currently, no population study has examined the association between night eating and all-cause, cancer and diabetes mortality risk.

There is a growing body of evidence suggesting that the level, type, and timing of food intake are all crucial factors in maintaining health [9]. Likewise, the health outcomes of eating during the night may be modified by factors such as timing, frequency, and food quality. Indeed, previous studies have found that the later eating times of the last meal were related to higher waist circumference and higher risk of type 2 diabetes mellitus [7, 10], yet no studies assessed the specific timing spectrum of night eating and their association with mortality risk. Higher daily eating frequency was associated with lower risk of all-cause mortality in people with diabetes [11], but it is unknown whether that relations would differ during the night. The higher percentage of energy intake at night was associated with an increased risk of type 2 diabetes mellitus or cancer [7, 12]. Conversely, nighttime consumption of small and low energy foods did not appear to be harmful and even showed a protective role in cardio-metabolic health [13, 14]. The conflicting results seems to indicate that a “correct” food quality can be advised to avoid the adverse effects of night eating. Nevertheless, no study has assessed the relationship between food quality of night eating and all-cause, cancer, and diabetes mortality risk.

In the current study, we analyzed the association between night eating (timing, frequency, and food quality) and the risks of all-cause, cancer, and diabetes mortality among 41,744 adults from the 2002-2018 US National Health and Nutrition Examination Survey (NHANES) data. We aimed to provide a comprehensive understanding of the potential health risks associated with night eating and offer insights into tailoring public health strategies for the reduction of excess mortality risk.

## Material and methods

The finding followed the Strengthening the Reporting of Observational Studies in Epidemiology guideline [15] (STROBE-NUT).

### Study population

The NHANES is a large periodic study that investigated the health and nutritional data from the US population. Details of the NHANES repository have been described elsewhere [16]. The study populations and all data were freely obtained from NHANES. The research ethics review board of the National Centre for Health Statistics Research approved the NHANES study, and all involved participants provided informed consent.

Participants who had one valid dietary questionnaire at baseline were included (*n* = 109,653). We excluded 33,018 individuals whose dietary recalls were unreliable and did not meet the minimum criteria for NHANES, 32,134 individuals with aged <20 years, 596 individuals who reported an energy intake of >5000 kcal/d or <500 kcal/d, 243 individuals who refused to answer and/or had missing mortality events, 784 individuals who lacked complete information on dietary intake, 1134 individuals who were pregnant at baseline. Overall, our sample consisted of 41,744 participants (Supplementary Fig. 1).

### Dietary assessment

Baseline dietary intake from 2002 to 2018 was obtained from the first 24-h dietary recall interview. The first recall was executed through personal interaction by proficient personnel at the NHANES mobile examination facilities. Standardized protocols and measuring tools were utilized to facilitate the evaluation of food volume and dimensions. During the interview, the participants were requested to provide information regarding the consumption quantity and time of each food and beverage item. The nutrient values were determined by utilizing the Food and Nutrient Database for Dietary Studies (FNDDS). The dietary intake of NHANES participants was integrated into 37 major groups of MyPyramid, as per the USDA’s Food Patterns Equivalents Database 2017–2018 (FPED, 2017–2018). The dietary supplements were obtained through the administration of a dietary supplement questionnaire. To assess diet quality, we calculated the Healthy Eating Index-2015 (HEI-2015), which is a measure for evaluating the alignment of foods with the American Dietary Guidelines for 2015–2020 Dietary Guidelines for Americans [17]. Dietary intake (without dietary supplement) was adjusted for total energy intake using the residual method.

### Main exposures

The main exposures were timing, frequency, and food quality of night eating. The night eating was defined as food consumption between 22:00 to 4:00 based on natural light cycle rhythm in this study. Timing of night eating was categorized into seven groups: “no eating“, “22:00 to 23:00“, “23:00 to 00:00“, “00:00 to 1:00“, “1:00 to 2:00“, “2:00 to 3:00“, and “3:00 to 4:00“; where if an individual had night eating more than twice a day, choose the timing of night eating based on the latest time point. Frequency of night eating was categorized into three groups: “no eating”, “one time”, and “two times and over”.

Food parameters of energy intake, low energy density foods intake (fruits, vegetables, whole grains, dairies, and protein foods), and high energy density foods intake (refined grains, add sugars, oils, solid fats, and alcoholic drinking) were used to generate a food quality for night eating. Each food parameter was preliminarily categorized by two levels and assigned into category 1 or 2 levels, respectively; where energy intake <200 kcal was assigned into 1, energy intake ≥200 kcal was assigned into 2; where food intakes <the median values of all foods intakes was assigned into 1, where food intakes ≥the median values of all foods intakes was assigned into 2. Then, based on above categories, food quality of night eating was defined using latent class analysis. A reasonable model was selected by analyzing latent classes with different numbers of latent classes. Akaike information criterion (AIC) and bayesian information criterion (BIC) were computed for the model selection (Supplementary Fig. 2) and four classes was selected (latent class 1, 2, 3, and 4). The item-response probabilities in models from four latent classes were shown in Supplementary Table 1.

Further, the characteristics of the four latent classes were assessed according to food intake in different latent class (Supplementary Table 2). Latent class 1 was characterized by very low energy intake, low intakes from low energy density foods, and very low intakes from high energy density foods, which could be labeled “very low dietary-energy-density intake” (VL-energy intake); latent class 2 was characterized by low energy intake, very low intakes from low energy density foods, and moderate intakes from high energy density foods, which could be labeled “low dietary-energy-density intake” (L-energy intake); latent class 3 was characterized by moderate energy intake, moderate intakes from low energy density foods, and moderate intakes from high energy density foods, which could be labeled “moderate dietary-energy-density intake” (M-energy intake); latent class 4 was characterized by high energy intake, high intakes from low energy density foods, and high intakes from high energy density foods, which could be labeled “high dietary-energy-density intake” (H-energy intake).

### Defining outcome

The outcomes were all-cause, cancer, and diabetes mortality that transpired subsequent to the survey participation date and prior to December 31, 2019. The National Death Index (NDI) was utilized to obtain death information. The NDI was publicly distributed by centers for disease control and prevention, Public-use Linked Mortality Files from National Center for Health Statistics, which are available for NHANES (National Center for Health Statistics. Office of Analysis and Epidemiology, Public-use Linked Mortality File, 2015. Hyattsville, Maryland. https://www.cdc.gov/nchs/data-linkage/mortality-public.htm). The International Classification of Diseases 10th Revision (ICD-10) was adopted to classify cause specific mortality; cancer mortality was identified by the ICD-10 codes C00-C9, and diabetes mortality was identified by the ICD-10 codes E10–E14. In total, 6066 deaths were recorded; of them, 1381 deaths were due to cancer and 206 deaths were due to diabetes.

### Covariates

Covariates included age (years), sex (male/female), race/ethnicity (Mexican American/non-Hispanic Black/non-Hispanic White/other Hispanic/other), education (less than 9th grade/9–11th grade/college graduate or above/high school graduate or GED or equivalent/some college or AA degree), income ($0–$19,999/$20,000–$44,999/$45,000–$74,999/$75,000–$99,999/ ≥ $100,000), smoking status (never smoker/past smoker/current smoker), drinking status (never drinker/past drinker/current drinker), body mass index (kg/m^2^), physical activity (metabolic equivalent hours per week (METs-h/week), sleep hours (hours/day), dietary energy intake (kcal), adherence to HEI-2015, dietary supplement use (%), glycohemoglobin (%), triglycerides (mmol/L), fasting glucose (mmol/L), total cholesterol (TCHO, mg/dL), oral glucose tolerance test (OGTT, mg/dL), hypertension, hyperlipidemia, cardiovascular disease (CVD), diabetes, and cancer. Drinking status was defined as never drinker (drank <12 drinks lifetime), past drinker (drank ≥12 drink lifetime and nondrinker over the past 12 months, and current drinker (drank ≥12 drink and currently a drinker). Smoking status was defined as never smoker (smoked <100 cigarettes lifetime), past smoker (smoked >100 cigarettes lifetime and currently did not smoke), and current smoker (smoked >100 cigarettes and currently a smoker). Timing of blood draw for biochemical variables detection was in the morning recorded by NHANES. Diabetes was defined as self-reported, diagnosed diabetes, hemoglobin A1c (HbA1c) ≥ 6.5%, or fasting plasma glucose ≥7.0 mmol/L. Hypertension was defined as diagnosed hypertension, taking antihypertensive drugs, systolic blood pressure ≥140 mm Hg, or diastolic blood pressure ≥90 mm Hg. Hyperlipidemia was defined as taking antihyperlipidemic drugs, triglycerides ≥150 mg/dL, total cholesterol ≥200 mg/dL, low density lipoprotein cholesterol ≥130 mg/dL, or high-density lipoprotein cholesterol <40 mg/dL for male and <50 mg/dL for female [18].

### Statistical analyses

Analyses were performed according to NHANES analytic guidelines, including sample weights, stratification, and clustering. Data analyses were conducted by R version 4.2.3 (the R Core Team). A two-sided *P* < 0.05 indicated statistical significance. The baseline characteristics were expressed as the means ± SD or numbers (percentages).

Weighted Cox proportional hazards (CPH) regression models were applied to evaluate the associations of the timing, frequency and food quality of night eating with all-cause, cancer, and diabetes mortality (no night eating as a reference). Results were expressed as adjusted hazard ratios [aHR] with 95% confidence intervals [CI]. We adjusted for baseline age and sex in model 1. We further adjusted for baseline education, race/ethnicity, family income, and body mass index in mode 2. Finally, we additionally adjusted for baseline dietary energy intake, alcohol consumption per day, smoking status, physical activity, histories of diabetes, hypertension, CVD, cancer, hyperlipidemia, adherence to the HEI-2015 score, and dietary supplement use in model 3. Percentage of missing values from covariates was less than 10% except for sleep hours (20.2%) (Supplementary Table 3). Chained equations (multivariate imputation) were used to impute missing values.

Subgroup analyses were performed in CPH models, categorized by baseline age (<65 and ≥65 years), sex, body mass index (>25, 25–29, and ≥30 kg/m^2^), smoking status (never smoker, past smoker, and current smoker), drinking status (never drinker, past drinker, and current drinker), HEI-2015 score (<70 and ≥70), sleep hours (<6 and ≥6 hours).

Linear regression analysis was used to investigate the correlation among timing, frequency and quality of night eating and biochemical variables (HbA1c, triglycerides, total cholesterol, OGTT, fasting glucose). Results were expressed as *β* value with 95% CIs. Three models (models 1, 2, and 3) were adjusted as described above except that model 3 was further adjusted for total length of fasting time. The fasting time is defined as the time (in hours) from not eating or drinking (except water) to venipuncture [19].

### Sensitivity analyses

Sensitivity analyses were performed to assay the robustness of the results. Firstly, we excluded 810 participants who had over 50% energy intake from night to reduce the influence of night eating syndrome on the results. Secondly, we added eating time (9:00–10:00) and re-ran CPH analyses. Moreover, we repeated the CPH analysis after further adjusting for sleep times.

## Results

### Baseline characteristics

The baseline characteristics of involved participants across timing, frequency and quality of night eating are shown in Tables 1–3. For timing of night eating, compared with people who had no night eating, those who had a later timing of night eating were more likely to be young, men, non-Hispanic black, less educated, middle-income earners, to be physically active, have less sleep hours and have higher dietary energy intake at baseline; they were less likely to be smokers, adhere to HEI scores, have lower dietary supplement use, and have a history of hyperlipidemia, hypertension, CVD, cancer and diabetes. The nadir for fasting glucose and OGTT levels occurred between 2:00 and 3:00, for total cholesterol level occurred between 2:00 and 3:00, whereas the peak for triglycerides occurred between 3:00 and 4:00. For frequency and food quality of night eating, compared with participants who had no night eating, those who had a higher frequency and dietary energy density food intake from night eating were more likely to be young, men, non-Hispanic black, have physically active, have less sleep hours, have higher dietary energy intake and have lower glycohemoglobin, total cholesterol and OGTT levels at baseline; they were less likely be smokers, and to have hyperlipidemia, hypertension, CVD, diabetes, and cancer, while less likely to adhere to HEI score and take dietary supplement only in participants with a higher eating frequency.

**Table 1:** Baseline characteristics of participation, categorized by timing of night eating.

Characteristics	Timing of night eating						
	No night eating	22:00 to 23:00	23:00 to 00:00	00:00 to 1:00	1:00 to 2:00	2:00 to 3:00	3:00 to 4:00
Participants, *n*	31362	6262	2354	588	417	375	386
Age (years)	48.97 ± 0.21	45.37 ± 0.35	41.76 ± 0.46	38.25 ± 0.75	39.41 ± 1.01	40.80 ± 1.01	44.31 ± 0.93
Female	16302 (52.0)	3014 (48.1)	1134 (48.2)	235 (40.0)	158 (37.9)	148 (39.5)	151 (39.1)
Race/ethnicity							
Mexican American	5471 (17.4)	895 (14.3)	321 (13.6)	70 (11.9)	44 (10.6)	41 (10.9)	53 (13.7)
Non-Hispanic Black	5963 (19.0)	1522 (24.3)	725 (30.8)	170 (28.9)	124 (29.7)	119 (31.7)	102 (26.4)
Non-Hispanic White	14541 (46.4)	2657 (42.4)	911 (38.7)	248 (42.2)	181 (43.4)	159 (42.4)	176 (45.6)
Other Hispanic	2635 (8.4)	558 (8.9)	187 (7.9)	36 (6.1)	25 (6.0)	15 (4.0)	22 (5.7)
Other	2752 (8.8)	630 (10.1)	210 (8.9)	64 (10.9)	43 (10.3)	41 (10.9)	33 (8.5)
Education							
Less than 9th grade	3922 (12.5)	538 (8.6)	164 (7.0)	31 (5.3)	21 (5.0)	16 (4.3)	24 (6.2)
9-11th grade	4426 (14.1)	909 (14.5)	365 (15.5)	78 (13.3)	54 (12.9)	54 (14.4)	66 (17.1)
College graduate or above	6956 (22.2)	1495 (23.9)	466 (19.8)	135 (23.0)	91 (21.8)	65 (17.3)	54 (14.0)
High school graduate/GED or equivalent	7328 (23.4)	1416 (22.6)	537 (22.8)	117 (19.9)	94 (22.5)	123 (32.8)	114 (29.5)
Some college or AA degree	8730 (27.8)	1904 (30.4)	822 (34.9)	227 (38.6)	157 (37.6)	117 (31.2)	128 (33.2)
Income							
$0 to $19,999	7052 (22.5)	1377 (22.0)	584 (24.8)	138 (23.5)	88 (21.1)	95 (25.3)	89 (23.1)
$20,000 to $44,999	9949 (31.7)	1987 (31.7)	758 (32.2)	168 (28.6)	121 (29.0)	114 (30.4)	108 (28.0)
$45,000 to $74,999	6270 (20.0)	1300 (20.8)	469 (19.9)	144 (24.5)	90 (21.6)	74 (19.7)	94 (24.4)
$75,000 to $99,999	4240 (13.5)	803 (12.8)	304 (12.9)	85 (14.5)	68 (16.3)	49 (13.1)	56 (14.5)
$100,000 and Over	3851 (12.3)	795 (12.7)	239 (10.2)	53 (9.0)	50 (12.0)	43 (11.5)	39 (10.1)
Smoking status							
Never smoker	17272 (55.1)	3300 (52.7)	1191 (50.6)	292 (49.7)	215 (51.6)	155 (41.3)	158 (40.9)
Past smoker	8187 (26.1)	1474 (23.5)	467 (19.8)	102 (17.3)	79 (18.9)	76 (20.3)	91 (23.6)
Current smoker	5903 (18.8)	1488 (23.8)	696 (29.6)	194 (33.0)	123 (29.5)	144 (38.4)	137 (35.5)
Drinking status^a^							
Never drinker	4728 (15.1)	871 (13.9)	258 (11.0)	49 (8.3)	42 (10.1)	31 (8.3)	36 (9.3)
Past drinker	5884 (18.8)	983 (15.7)	345 (14.7)	70 (11.9)	41 (9.8)	56 (14.9)	60 (15.5)
Current drinker	20750 (66.2)	4408 (70.4)	1751 (74.4)	469 (79.8)	334 (80.1)	288 (76.8)	290 (75.1)
Body mass index (kg/m^2^)	28.85 ± 0.08	28.85 ± 0.13	29.00 ± 0.24	28.08 ± 0.39	27.68 ± 0.49	28.08 ± 0.45	29.49 ± 0.51
Physical activity (METs-h/week)	8.58 ± 0.07	8.67 ± 0.12	9.18 ± 0.19	9.10 ± 0.34	9.47 ± 0.42	9.45 ± 0.45	9.27 ± 0.47
Sleep hours (h/day)	7.13 ± 0.02	6.96 ± 0.03	7.10 ± 0.12	6.70 ± 0.09	6.96 ± 0.14	6.64 ± 0.12	6.56 ± 0.13
Dietary energy intake (kcal)	2015.49 ± 7.15	2220.85 ± 15.00	2274.07 ± 27.02	2548.01 ± 45.64	2595.00 ± 68.40	2586.35 ± 78.46	2412.60 ± 61.00
Adherence to HEI-2015 score	51.14 ± 0.18	50.12 ± 0.28	48.19 ± 0.40	49.55 ± 0.72	49.11 ± 0.85	48.47 ± 1.48	46.91 ± 0.86
Dietary supplement use (%)	11663 (37.2)	2077 (33.2)	633 (26.9)	146 (24.8)	132 (31.7)	108 (28.8)	118 (30.6)
Glycohemoglobin (%)	5.60 ± 0.01	5.59 ± 0.02	5.60 ± 0.02	5.49 ± 0.06	5.57 ± 0.06	5.47 ± 0.05	5.57 ± 0.06
Triglycerides (mmol/L)	1.73 ± 0.01	1.74 ± 0.03	1.64 ± 0.04	1.61 ± 0.06	1.68 ± 0.11	1.65 ± 0.10	1.93 ± 0.10
Fasting glucose (mmol/L)	5.89 ± 0.01	5.89 ± 0.03	5.94 ± 0.05	5.83 ± 0.10	5.84 ± 0.10	5.61 ± 0.07	5.80 ± 0.09
Total cholesterol (mmol/L)	5.11 ± 0.01	5.03 ± 0.02	5.00 ± 0.03	4.94 ± 0.06	5.03 ± 0.08	4.92 ± 0.11	5.14 ± 0.07
OGTT (mg/dL)	133.60 ± 0.64	130.59 ± 1.35	127.01 ± 1.81	123.16 ± 4.22	121.49 ± 4.06	112.02 ± 3.88	125.12 ± 4.10
Fasting time (h)	7.60 ± 0.06	7.23 ± 0.10	6.35 ± 0.13	7.47 ± 0.26	7.04 ± 0.35	6.88 ± 0.34	7.19 ± 0.31
Hyperlipidemia	22516 (71.8)	4196 (67.0)	1425 (60.5)	319 (54.3)	243 (58.3)	222 (59.2)	233 (60.4)
Hypertension	13437 (42.9)	2427 (38.8)	847 (36.0)	161 (27.4)	155 (37.2)	129 (34.4)	147 (38.1)
CVD	10698 (34.1)	1952 (31.2)	624 (26.5)	126 (21.4)	100 (24.0)	104 (27.7)	120 (31.1)
Diabetes	5870 (18.7)	1140 (18.2)	363 (15.4)	63 (10.7)	45 (10.8)	44 (11.7)	59 (15.3)
Cancer	3298 (10.5)	509 (8.1)	170 (7.2)	34 (5.8)	29 (7.0)	20 (5.3)	28 (7.3)

*HEI-2015* Healthy Eating Index 2015, *CVD* cardiovascular disease, *METs-h* metabolic equivalent hours, *OGTT* oral glucose tolerance test.

^a^Mild drinking: one drinking for female and two drinking for male; moderate drinking: two drinking for female and three drinking for male, or binge ≥2 & binge <5; heavy drinking: three drinking for female and four drinking for male, or binge ≥5. Continuous variables were adjusted for survey weights of NHANES. Categorical variables were unweighted.

**Table 2:** Baseline characteristics of participation, categorized by night eating frequency.

Characteristics	Night eating frequency		
	No night eating	One time	Two times and over
Participants, *n*	31,362	9050	1332
Age (years)	48.97 ± 0.21	44.38 ± 0.29	39.00 ± 0.56
Female	16302 (52.0)	4305 (47.6)	535 (40.2)
Race/ethnicity			
Mexican American	5471 (17.4)	1269 (14.0)	155 (11.6)
Non-Hispanic Black	5963 (19.0)	2325 (25.7)	437 (32.8)
Non-Hispanic White	14541 (46.4)	3810 (42.1)	522 (39.2)
Other Hispanic	2635 (8.4)	754 (8.3)	89 (6.7)
Other	2752 (8.8)	892 (9.9)	129 (9.7)
Education			
Less than 9th grade	3922 (12.5)	730 (8.1)	64 (4.8)
9–11th grade	4426 (14.1)	1338 (14.8)	188 (14.1)
College graduate or above	6956 (22.2)	2043 (22.6)	263 (19.7)
High school graduate/GED or equivalent	7328 (23.4)	2068 (22.9)	333 (25.0)
Some college or AA degree	8730 (27.8)	2871 (31.7)	484 (36.3)
Income			
$0 to $19,999	7052 (22.5)	2058 (22.7)	313 (23.5)
$20,000 to $44,999	9949 (31.7)	2856 (31.6)	400 (30.0)
$45,000 to $74,999	6270 (20.0)	1888 (20.9)	283 (21.2)
$75,000 to $99,999	4240 (13.5)	1162 (12.8)	203 (15.2)
$100,000 and over	3851 (12.3)	1086 (12.0)	133 (10.0)
Smoking status			
Never smoker	17272 (55.1)	4687 (51.8)	624 (46.8)
Past smoker	8187 (26.1)	2035 (22.5)	254 (19.1)
Current smoker	5903 (18.8)	2328 (25.7)	454 (34.1)
Drinking status^a^			
Never drinker	4728 (15.1)	1173 (13.0)	114 (8.6)
Past drinker	5884 (18.8)	1386 (15.3)	169 (12.7)
Current drinker	20750 (66.2)	6491 (71.7)	1049 (78.8)
Body mass index (kg/m^2^)	28.85 ± 0.08	28.87 ± 0.11	28.18 ± 0.29
Physical activity (METs-h/week)	8.58 ± 0.07	8.82 ± 0.11	9.41 ± 0.23
Sleep hours (h/day)	7.13 ± 0.02	6.94 ± 0.03	7.01 ± 0.16
Dietary energy intake (kcal)	2015.49 ± 7.15	2240.98 ± 12.11	2619.97 ± 36.04
Adherence to HEI-2015 score	51.14 ± 0.18	49.56 ± 0.26	48.55 ± 0.52
Dietary supplement use (%)	11663 (37.2)	2866 (31.7)	348 (26.1)
Glycohemoglobin (%)	5.60 ± 0.01	5.59 ± 0.01	5.52 ± 0.03
Triglycerides (mmol/L)	1.73 ± 0.01	1.72 ± 0.03	1.65 ± 0.06
Fasting glucose (mmol/L)	5.89 ± 0.01	5.89 ± 0.02	5.80 ± 0.06
Total cholesterol (mmol/L)	5.11 ± 0.01	5.02 ± 0.02	5.00 ± 0.05
OGTT (mg/dL)	133.60 ± 0.64	129.27 ± 1.08	119.45 ± 2.54
Fasting time (h)	7.60 ± 0.06	7.06 ± 0.08	6.78 ± 0.20
Hyperlipidemia	22516 (71.8)	5889 (65.1)	749 (56.2)
Hypertension	13437 (42.9)	3438 (38.0)	428 (32.1)
CVD	10698 (34.1)	2711 (30.0)	315 (23.6)
Diabetes	5870 (18.7)	1575 (17.4)	139 (10.4)
Cancer	3298 (10.5)	707 (7.8)	83 (6.2)

*HEI-2015* Healthy Eating Index 2015, *CVD* cardiovascular disease, *METs-h* metabolic equivalent hours, *OGTT* oral glucose tolerance test.

^a^Mild drinking: one drinking for female and two drinking for male; moderate drinking: two drinking for female and three drinking for male, or binge ≥2 & binge <5; heavy drinking: three drinking for female and four drinking for male, or binge ≥5. Continuous variables were adjusted for survey weights of NHANES. Categorical variables were unweighted.

**Table 3:** Baseline characteristics of the study population, categorized by food quality.

Characteristics	Food quality				
	No night eating	VL-energy intake	L-energy intake	M-energy intake	H-energy intake
Participants, *n*	31362	3113	1218	1868	4183
Age (years)	48.97 ± 0.21	48.04 ± 0.43	43.96 ± 0.62	42.39 ± 0.51	40.92 ± 0.35
Female	16302 (52.0)	1620 (52.0)	647 (53.1)	779 (41.7)	1794 (42.9)
Race/ethnicity					
Mexican American	5471 (17.4)	440 (14.1)	175 (14.4)	242 (13.0)	567 (13.6)
Non-Hispanic Black	5963 (19.0)	601 (19.3)	341 (28.0)	517 (27.7)	1303 (31.1)
Non-Hispanic White	14541 (46.4)	1447 (46.5)	505 (41.5)	725 (38.8)	1655 (39.6)
Other Hispanic	2635 (8.4)	270 (8.7)	110 (9.0)	163 (8.7)	300 (7.2)
Other	2752 (8.8)	355 (11.4)	87 (7.1)	221 (11.8)	358 (8.6)
Education					
Less than 9th grade	3922 (12.5)	271 (8.7)	102 (8.4)	143 (7.7)	278 (6.6)
9–11th grade	4426 (14.1)	397 (12.8)	181 (14.9)	269 (14.4)	679 (16.2)
College graduate or above	6956 (22.2)	854 (27.4)	224 (18.4)	460 (24.6)	768 (18.4)
High school graduate/GED or equivalent	7328 (23.4)	649 (20.8)	277 (22.7)	411 (22.0)	1064 (25.4)
Some college or AA degree	8730 (27.8)	942 (30.3)	434 (35.6)	585 (31.3)	1394 (33.3)
Income					
$0 to $19,999	7052 (22.5)	649 (20.8)	289 (23.7)	459 (24.6)	974 (23.3)
$20,000 to $44,999	9949 (31.7)	955 (30.7)	363 (29.8)	570 (30.5)	1368 (32.7)
$45,000 to $74,999	6270 (20.0)	649 (20.8)	253 (20.8)	384 (20.6)	885 (21.2)
$75,000 to $99,999	4240 (13.5)	423 (13.6)	188 (15.4)	224 (12.0)	530 (12.7)
$100,000 and over	3851 (12.3)	437 (14.0)	125 (10.3)	231 (12.4)	426 (10.2)
Smoking status					
Never smoker	17272 (55.1)	1639 (52.7)	627 (51.5)	951 (50.9)	2094 (50.1)
Past smoker	8187 (26.1)	799 (25.7)	259 (21.3)	436 (23.3)	795 (19.0)
Current smoker	5903 (18.8)	675 (21.7)	332 (27.3)	481 (25.7)	1294 (30.9)
Drinking status^a^					
Never drinker	4728 (15.1)	438 (14.1)	155 (12.7)	209 (11.2)	485 (11.6)
Past drinker	5884 (18.8)	476 (15.3)	197 (16.2)	300 (16.1)	582 (13.9)
Current drinker	20750 (66.2)	2199 (70.6)	866 (71.1)	1359 (72.8)	3116 (74.5)
Body mass index (kg/m^2^)	28.85 ± 0.08	28.77 ± 0.19	28.69 ± 0.27	28.76 ± 0.23	28.83 ± 0.18
Physical activity (METs-h/week)	8.58 ± 0.07	8.41 ± 0.13	8.74 ± 0.22	9.51 ± 0.23	9.02 ± 0.16
Sleep hours (hours/day)	7.13 ± 0.02	7.03 ± 0.06	6.99 ± 0.08	6.88 ± 0.05	6.91 ± 0.06
Dietary energy intake (kcal)	2015.49 ± 7.15	2216.12 ± 20.16	2160.04 ± 34.08	2469.47 ± 25.65	2302.13 ± 19.81
Adherence to HEI-2015 score	51.14 ± 0.18	51.83 ± 0.40	47.30 ± 0.53	52.12 ± 0.45	47.03 ± 0.32
Dietary supplement use (%)	11663 (37.2)	541 (29.0)	1232 (39.6)	348 (28.6)	1093 (26.1)
Glycohemoglobin (%)	5.60 ± 0.01	5.64 ± 0.02	5.54 ± 0.03	5.58 ± 0.03	5.56 ± 0.02
Triglycerides (mmol/L)	1.73 ± 0.01	1.70 ± 0.03	1.71 ± 0.06	1.83 ± 0.09	1.67 ± 0.03
Fasting glucose (mmol/L)	5.89 ± 0.01	5.97 ± 0.04	5.81 ± 0.06	5.87 ± 0.05	5.84 ± 0.03
Total cholesterol (mg/dL)	5.11 ± 0.01	5.02 ± 0.02	5.10 ± 0.05	5.00 ± 0.04	5.00 ± 0.02
OGTT (mg/dL)	133.60 ± 0.64	134.36 ± 1.85	127.81 ± 2.98	126.16 ± 2.31	124.12 ± 1.52
Fasting time (hours)	7.71 ± 0.06	6.93 ± 0.12	7.27 ± 0.21	7.12 ± 0.18	7.22 ± 0.11
Hyperlipidemia	22516 (71.8)	2136 (68.6)	792 (65.0)	1161 (62.2)	2549 (60.9)
Hypertension	13437 (42.9)	1301 (41.8)	470 (38.6)	662 (35.4)	1433 (34.3)
CVD	10698 (34.1)	1121 (36.0)	376 (30.9)	492 (26.3)	1037 (24.8)
Diabetes	5870 (18.7)	615 (19.8)	194 (15.9)	287 (15.4)	618 (14.8)
Cancer	3298 (10.5)	318 (10.2)	87 (7.1)	128 (6.9)	257 (6.1)

*HEI-2015* Healthy Eating Index 2015, *CVD* cardiovascular disease, *METs-h* metabolic equivalent hours, *OGTT* oral glucose tolerance test.

^a^Mild drinking: one drinking for female and two drinking for male; moderate drinking: two drinking for female and three drinking for male, or binge ≥2 & binge <5; heavy drinking: three drinking for female and four drinking for male, or binge ≥5. Continuous variables were adjusted for survey weights of NHANES. Categorical variables were unweighted.

### The association between timing, frequency and quality of night eating and mortality

**Fig. 1:**
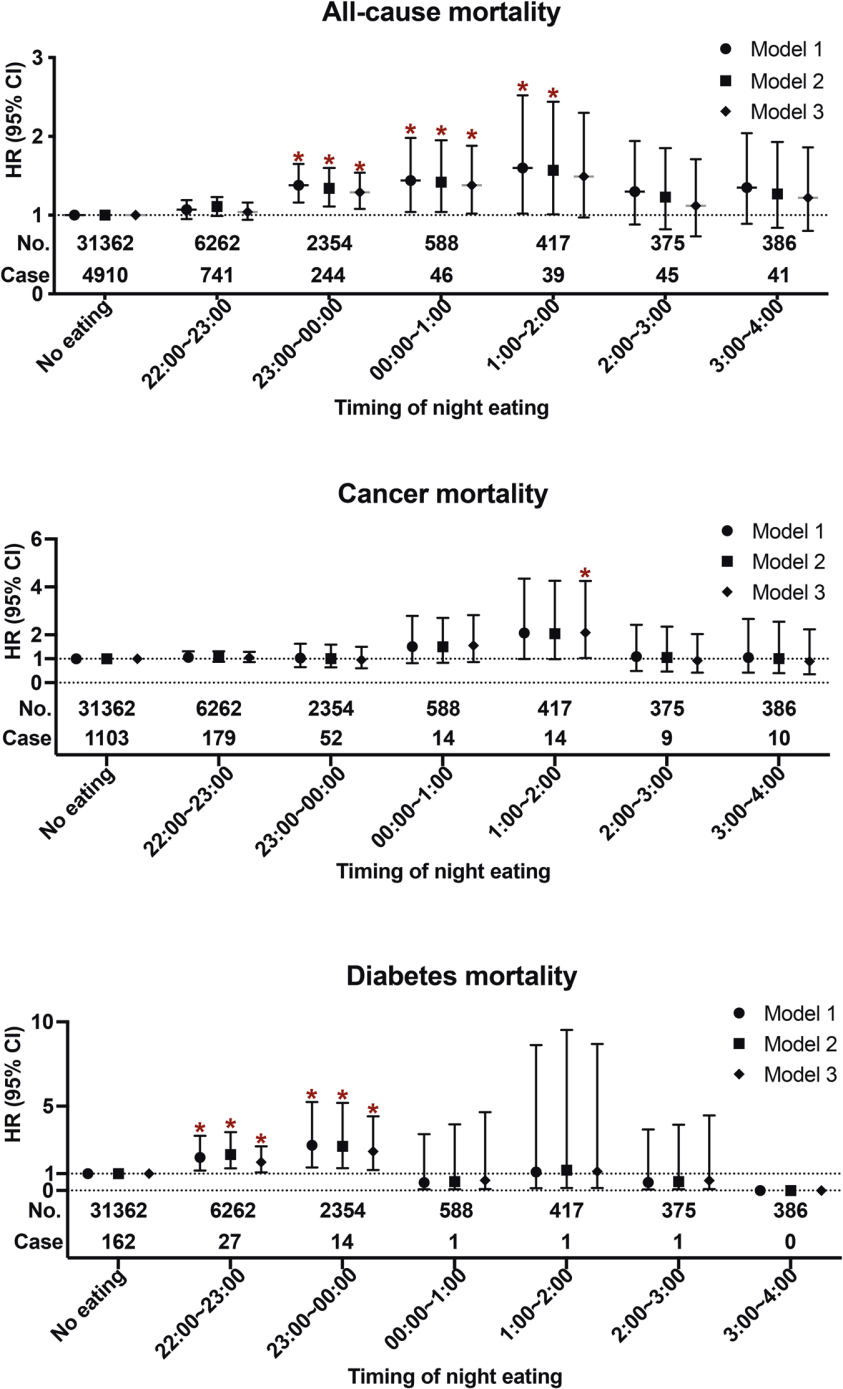
Associations between timing of night eating with all-cause, cancer, and diabetes mortality. aHR adjusted hazard ratio, CI confidence intervals, HEI-2015 Healthy Eating Index 2015, CVD cardiovascular disease. *aHR (95%CI) was estimated by weighted Cox regression analyses. Date is shown as aHR with 95%CI. *Represented the significant association between timing of night eating and mortality. Model 1 adjusted for age and sex. Model 2 further adjusted for education, race/ethnicity, family income, and BMI. Model 3 further adjusted for dietary energy intake, drinking status, smoking status, physical activity, diabetes, hypertension, hyperlipidemia, CVD, cancer, adherence to HEI-2015 score, and dietary supplement use.

Among 41744 participants, 6066 deaths occurred during a median follow-up of 8.7 years, including 1381 deaths from cancer and 206 from diabetes. The association of timing night eating with all-cause, cancer and diabetes mortality were first evaluated (Fig. 1 and Supplementary Table 4). Compared with people who had no night eating, individuals who had later timing of night eating were positively associated with higher risk of all-cause and diabetes mortality in model 1 of adjusting age and sex (all-cause mortality: *P*-trend <0.001; diabetes mortality: *P*-trend =0.016). This association remained significant after further adjusting for education, race/ethnicity, family income, and BMI in model 2. In model 3 of further adjusting for multiple risk factors, this estimate for all-cause mortality risk was attenuated but continued to exist (*P*-trend = 0.002), and the timing of 1:00-2:00 had the highest risk trend (aHR 1.49 [95% CI 0.97–2.30]), followed by 00:00–1:00 (aHR 1.38 [95% CI 1.02-1.88]), 23:00–00:00 (aHR 1.29 [95% CI 1.08–1.54]), 3:00–4:00 (aHR 1.22 [95% CI 0.80–1.86]), while non-significant for the timing of 2:00–3:00 (aHR 1.12 [95% CI 0.73–1.71]), and 22:00-23:00 (aHR 1.04 [95% CI 0.94–1.16]). For diabetes mortality, compared with no night eating, night eating was associated with 1.68-fold (aHR 1.68 [95% CI 1.07-2.62]) and 2.31-fold (aHR 2.31 [95% CI 1.21-4.40]) higher mortality risk for the timing of 22:00–23:00 and 23:00–00:00, respectively. No statistical significance between timing of night eating and risk of cancer mortality was detected in the adjusted models (each *P*-trend >0.05), except that aHR for the timing of eating from 1:00 to 2:00 was 2.09 (1.03–4.25), as compared with no night eating.

**Fig. 2:**
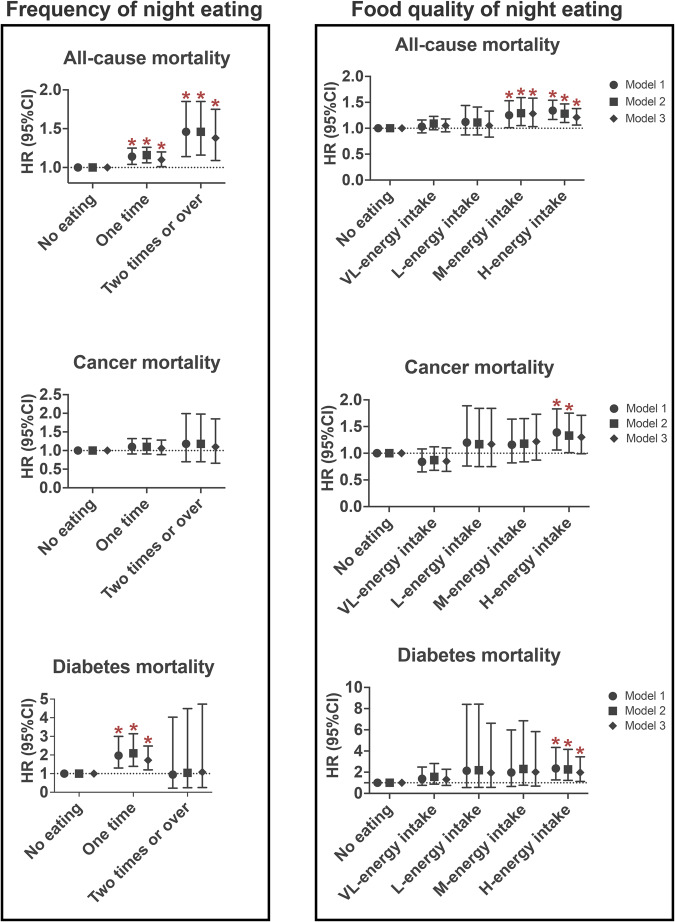
Association between frequency and food quality of night eating with all-cause, cancer, and diabetes mortality. aHR adjusted hazard ratio, CI confidence intervals, HEI-2015 Healthy Eating Index 2015, CVD cardiovascular disease. *aHR (95%CI) was estimated by weighted Cox regression analyses. Date is shown as aHR with 95%CI. *Represented the significant association between timing of night eating and mortality. Model 1 adjusted for age and sex. Model 2 further adjusted for education, race/ethnicity, family income, and BMI. Model 3 further adjusted for dietary energy intake, drinking status, smoking status, physical activity, diabetes, hypertension, hyperlipidemia, CVD, cancer, adherence to HEI-2015 score, and dietary supplement use.

The association of frequency of night eating with all-cause, cancer and diabetes mortality were also assessed (Fig. 2 and Supplementary Table 5). In models 1, compared with no night eating, more frequent night eating behavior was associated with higher all-cause and diabetes mortality risk (all-cause mortality: *P*-trend <0.001; diabetes mortality: *P*-trend = 0.007), but not cancer mortality; the same was true for model 2. In multivariate-adjusted model 3, compared with individuals who had no night eating, participants who had more frequent night eating still had a significantly higher risk of all-cause and/or diabetes mortality; the aHR of all-cause and diabetes mortality was 1.10 (95% CI 1.01–1.20) and 1.72 (95% CI 1.20–2.48) for one time, respectively, and 1.38 (95% CI 1.09–1.75) for two times or over.

The associations between food quality of night eating and mortality risk were further examined (Fig. 2 and Supplementary Table 6). The poorer dietary quality characterized by higher dietary energy density intake from night eating was associated with a higher risk of all-cause, cancer and diabetes mortality in all models (each *P*-trend <0.05). Compared with the no night eating group, the H-energy intake group was significantly and nominally associated with a 1.21-fold, 1.30-fold and 1.97-fold increased risk for all-cause mortality (aHR 1.21 [95% CI 1.06–1.38]), cancer mortality (aHR 1.30 [95% CI 0.99–1.71]), and diabetes mortality (aHR 1.97 [95% CI 1.13–3.45]), respectively; the M-energy intake group was associated with a 1.28-fold increased risk (aHR 1.28 [95% CI 1.03–1.58]) for all-cause mortality and had a high risk trend toward cancer mortality (aHR 1.22 [95% CI 0.87–1.73]) and diabetes mortality (aHR 2.01 [95% CI 0.69–5.83]); nevertheless, the VL-energy intake and L-energy intake group showed no association.

### Subgroup analysis

Furthermore, subgroup analyses were performed (Supplementary Tables 7–9). Subgroup analysis revealed that age, sex, body mass index, smoking status, drinking status, HEI-2015 score, and sleep hours did not impact the association between timing, frequency and food quality of night eating with all-cause, cancer and diabetes mortality (each *P*-interaction > 0.05).

### The association of timing, frequency and quality of night eating and serum biochemical variables

**Fig. 3:**
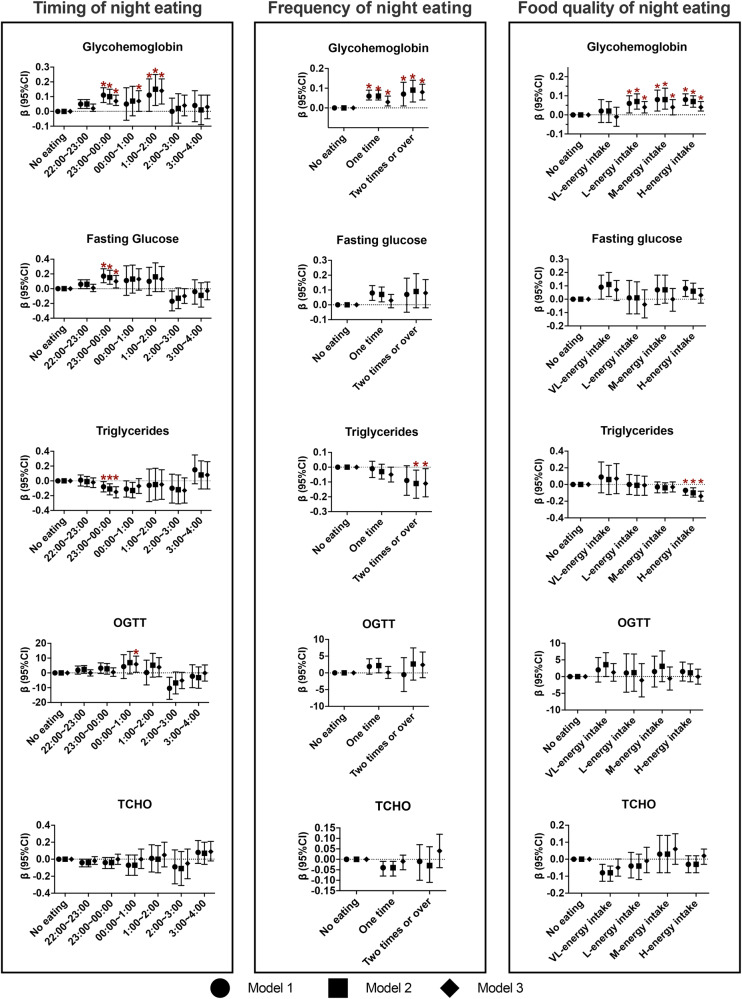
Association of night eating in timing, frequency and food quality with serum biochemical variables. CI confidence intervals, HEI-2015 Healthy Eating Index 2015, CVD cardiovascular disease. *Represented the significant association. Model 1 adjusted for age and sex. Model 2 further adjusted for education, race/ethnicity, family income, and BMI. Model 3 further adjusted for dietary energy intake, drinking status, smoking status, physical activity, diabetes, hypertension, hyperlipidemia, CVD, cancer, adherence to HEI-2015 score, and dietary supplement use, and total length of fasting time.

Next, weighed linear regression analysis was applied to investigate the relationship between timing, frequency and quality of night eating and biochemical variables (Fig. 3). Inverse correlations were observed between triglycerides and night eating from the timing of 23:00–00:00, from the frequency of eating two or more times, and from the H-energy intake group. In contrast, glycohemoglobin had positive correlations with night eating from the timing of 23:00–00:00, 00:00–1:00, and 1:00–2:00, from the frequency of eating one or more times, and from the L-energy intake, M-energy intake, and H-energy intake groups. Significant positive correlations were observed among fasting glucose, OGTT and night eating from the timing of 23:00–00:00 and 00:00–1:00, respectively, while null associations were observed among fasting glucose, OGTT and night eating in frequency and food quality. TCHO was not related with night eating in timing, frequency and food quality.

### Sensitivity analysis

In sensitivity analyses, the results did not materially change when further adjusting for sleep times (Supplementary Tables 4–6), or including the time period of night eating from 9:00 to 10:00 (Supplementary Tables 10–12). Moreover, similar results were still found after further excluding participants whose nocturnal energy intake exceeds 50% (Supplementary Tables 13–15).

## Discussion

In this study, compared with no night eating, we found the following: (i) the later timing of night eating was associated with increased risks of all-cause and diabetes mortality, with the significant risk of eating between 23:00 and 1:00, and between 22:00 and 00:00, respectively; (ii) more frequent night eating exposure was significantly associated with higher risks of all-cause and diabetes mortality; (iii) night eating from M-energy and H-energy intake groups were associated with increased risk of all-cause, cancer and diabetes mortality, but this association was not observed from the VL-energy intake and L-energy intake groups; (iv) partially align with the mortality risk, night eating had positive correlations with glycohemoglobin, fasting glucose or OGTT, and an inverse correlation with triglycerides, depending on the distinct timing, frequency and food quality of night eating.

Previous observational studies have shown that the later timing of eating was related to overweight/obese [10, 20, 21]. The later timing of eating was also associated with higher risk of type 2 diabetes mellitus from the Korea National Health and Nutrition Survey [7]. Our study extended those previous reports concerning the timing of night eating and is the first, to our knowledge, to focus on the specific timing spectrum during night eating period and their associations with all-cause, cancer and diabetes mortality. We revealed that, compared with no night eating, night eating was associated with increased all-cause mortality only for eating between 23:00 and 1:00, cancer mortality only for eating between 1:00 and 2:00, and diabetes mortality for eating between 22:00 and 00:00. It is interesting to note that food consumption between 9:00 and 22:00 showed no significant association with mortality risk. That results may indicate that if we had a night eating habit, the timing of food consumption before 22:00 would be conservatively suggested.

Our study also found that, compared with no night eating, more frequent night eating was associated with higher risks of all-cause and diabetes mortality, but not with cancer mortality. Inconsistent with the results, the idea that a higher daily eating frequency might be beneficial for health has been suggested in previous studies [10–12, 22–24]. Findings from NHANES found that higher daily eating frequency was associated with lower risk of all-cause mortality in people with diabetes or general adults [11, 25]. Another study found that the higher eating frequency in a day was associated with lower risk of cancer in women [12]. More other studies showed that a higher daily eating frequency was related to robust circadian rhythms and/or lower risk of metabolic syndrome [10, 23, 24]. We speculated that the inconsistent results may be due to the differences between daytime and nights. Thus, high frequency should be avoided when food consumption occurs during the night.

We further found that, compared with no night eating, night eating with poorer food quality characterized by higher dietary energy density was associated with higher risk of all-cause, cancer and diabetes mortality; however, night eating with low dietary-energy-density intake (the average energy intake ≤342.91 kcal) was not related to all-cause, cancer and diabetes mortality. The above observations are partially supported by a series of previous studies [7, 12–14, 26]. First, the high energy intake at night was positively associated with the risk of type 2 diabetes mellitus or cancer [7, 12], while small and low energy foods from night eating showed a protective role in muscle protein synthesis or cardiometabolic health [13, 14, 26]. Furthermore, high dietary energy density is positively associated with type 2 diabetes [27, 28] and cancers [29, 30]. Obviously, people who had night eating habit should be strongly advised against high dietary energy density foods.

The possible mechanism connecting night eating and high mortality risk might involve unhealthy food intake [31], higher energy intake [32], disrupted circadian rhythms [18, 33, 34], and disrupted glucose and lipid metabolism [35–37]. Circadian disruption has been shown to have a higher mortality risk, trigger the onset of diabetes, and drive tumor progression [38, 39]; in turn, circadian alignment by modulating the timing of food intake promoted health benefits [9, 40–42]. Therefore, we speculated that night eating may disrupt circadian rhythm, probably contributing to high mortality risk. We also found that significant positive correlations between glycohemoglobin, fasting glucose, and/or OGTT and night eating. Our results are supported by epidemiological evidence showing that glycohemoglobin, fasting glucose, or OGTT were associated with increased all-cause or cancer mortality with or without diabetes [43–45]. However, a negative correlation between triglycerides and night eating was observed, which is inconsistent with finding showing that elevated blood triglycerides levels were associated with higher all-cause mortality risk [46]. Possible explanation may involve in negative feedback of triglycerides metabolism during night eating, however, the mechanisms for this merit exploration further.

This study had several major strengths. First, this is the first study exploring the association between timing, frequency and food quality of night eating and mortality risk. Another strength is that we adjusted the analyses for a broad range of confounders, including traditional and novel risk factors. Additionally, this study was derived from well-designed and nationally representative NHANES samples, thus making our results more generalizable and repeatable.

Our study also had some limitations. First, dietary information was collected by 24-h dietary recalls and the night eating information was only evaluated at the baseline, which may not fully reflect long-term eating habits. Second, baseline dietary intake was merely obtained from the first 24-h dietary recall interview because specific timing of night eating from the first 24-h was more accurate than the second 24-h dietary recall. Third, although major confounders were adjusted for in CPH regression models, unknown and unmeasured confounding likely exist. Fourth, we were unable to obtain the specific sleeping time and career information, would have impact the association of night eating with mortality risk, although sleep duration is adjusted for in this study. Lastly, mortality events were rarely reported when we assessed the relationship between timing of night eating and diabetes mortality, so some results became weaken.

## Conclusions

In conclusion, night eating was significantly associated with increased all-cause, cancer and diabetes mortality, with varying timing, frequency and food quality. These findings highlight that eating before 23:00 or low dietary-energy-density foods could be suggested for the reduction of excess mortality risk during night eating.

### Supplementary information

Supplementary materials

## Data Availability

The data of this article will be shared on reasonable request to the corresponding author.

## References

[1] Dashti HS, Scheer F, Saxena R, Garaulet M (2019). Timing of food intake: identifying contributing factors to design effective interventions. Adv Nutr..

[2] Hawley JA, Sassone-Corsi P, Zierath JR (2020). Chrono-nutrition for the prevention and treatment of obesity and type 2 diabetes: from mice to men. Diabetologia..

[3] Wang P, Jiang X, Tan Q, Du S, Shi D (2023). Meal timing of dietary total antioxidant capacity and its association with all-cause, CVD and cancer mortality: the US national health and nutrition examination survey, 1999-2018. Int J Behav Nutr Phys Act..

[4] Li M, Tse LA, Chan WC, Kwok CH, Leung SL, Wu C (2017). Nighttime eating and breast cancer among Chinese women in Hong Kong. Breast Cancer Res..

[5] Kogevinas M, Espinosa A, Castello A, Gomez-Acebo I, Guevara M, Martin V (2018). Effect of mistimed eating patterns on breast and prostate cancer risk (MCC-Spain Study). Int J Cancer..

[6] Hood MM, Reutrakul S, Crowley SJ (2014). Night eating in patients with type 2 diabetes. Associations with glycemic control, eating patterns, sleep, and mood. Appetite..

[7] Kwak J, Jang KA, Kim HR, Kang MS, Lee KW, Shin D (2023). Identifying the associations of nightly fasting duration and meal timing with type 2 diabetes mellitus using data from the 2016-2020 Korea National Health and Nutrition Survey. Nutrients..

[8] Wang C, Almoosawi S, Palla L (2021). Relationships between food groups and eating time slots according to diabetes status in adults from the UK National Diet and Nutrition Survey (2008-2017). Front Nutr..

[9] Longo VD, Anderson RM (2022). Nutrition, longevity and disease: from molecular mechanisms to interventions. Cell..

[10] Makarem N, Sears DD, St-Onge MP, Zuraikat FM, Gallo LC, Talavera GA (2020). Habitual nightly fasting duration, eating timing, and eating frequency are associated with cardiometabolic risk in women. Nutrients..

[11] Xie J, Wang Z, Zhang X, Wang J, Feng W, Hu Y (2023). Association between daily eating frequency and mortality in people with diabetes: findings from NHANES 1999-2014. Front Nutr..

[12] Marinac CR, Sears DD, Natarajan L, Gallo LC, Breen CI, Patterson RE (2015). Frequency and circadian timing of eating may influence biomarkers of inflammation and insulin resistance associated with breast cancer risk. PLoS One..

[13] Kinsey AW, Eddy WR, Madzima TA, Panton LB, Arciero PJ, Kim JS (2014). Influence of night-time protein and carbohydrate intake on appetite and cardiometabolic risk in sedentary overweight and obese women. Br J Nutr..

[14] Kinsey AW, Ormsbee MJ (2015). The health impact of nighttime eating: old and new perspectives. Nutrients..

[15] Lachat C, Hawwash D, Ocke MC, Berg C, Forsum E, Hornell A (2016). Strengthening the Reporting of Observational Studies in Epidemiology-Nutritional Epidemiology (STROBE-nut): an extension of the STROBE statement. PLoS Med..

[16] Shan Z, Rehm CD, Rogers G, Ruan M, Wang DD, Hu FB (2019). Trends in dietary carbohydrate, protein, and fat intake and diet quality among US adults, 1999-2016. JAMA..

[17] Krebs-Smith SM, Pannucci TE, Subar AF, Kirkpatrick SI, Lerman JL, Tooze JA (2018). Update of the Healthy Eating Index: HEI-2015. J Acad Nutr Diet..

[18] Kammerlander AA, Mayrhofer T, Ferencik M, Pagidipati NJ, Karady J, Ginsburg GS (2021). Association of metabolic phenotypes with coronary artery disease and cardiovascular events in patients with stable chest pain. Diabetes Care..

[19] Wirth MD, Zhao L, Turner-McGrievy GM, Ortaglia A (2021). Associations between Fasting Duration, Timing of First and Last Meal, and Cardiometabolic Endpoints in the National Health and Nutrition Examination Survey. Nutrients.

[20] McHill AW, Phillips AJ, Czeisler CA, Keating L, Yee K, Barger LK (2017). Later circadian timing of food intake is associated with increased body fat. Am J Clin Nutr..

[21] Martinez-Lozano N, Tvarijonaviciute A, Rios R, Baron I, Scheer F, Garaulet M (2020). Late eating is associated with obesity, inflammatory markers and circadian-related disturbances in school-aged children. Nutrients..

[22] Vilela S, Severo M, Moreira T, Oliveira A, Hetherington MM, Lopes C (2019). Association between eating frequency and eating behaviours related to appetite from 4 to 7 years of age: findings from the population-based birth cohort generation XXI. Appetite..

[23] Garciduenas-Fimbres TE, Paz-Graniel I, Nishi SK, Salas-Salvado J, Babio N (2021). Eating speed, eating frequency, and their relationships with diet quality, adiposity, and metabolic syndrome, or its components. Nutrients..

[24] Zeron-Rugerio MF, Diez-Noguera A, Izquierdo-Pulido M, Cambras T (2021). Higher eating frequency is associated with lower adiposity and robust circadian rhythms: a cross-sectional study. Am J Clin Nutr..

[25] Chen HJ, Wang Y, Cheskin LJ (2016). Relationship between frequency of eating and cardiovascular disease mortality in U.S. adults: the NHANES III follow-up study. Ann Epidemiol..

[26] Groen BB, Res PT, Pennings B, Hertle E, Senden JM, Saris WH (2012). Intragastric protein administration stimulates overnight muscle protein synthesis in elderly men. Am J Physiol Endocrinol Metab..

[27] Hingle MD, Wertheim BC, Neuhouser ML, Tinker LF, Howard BV, Johnson K (2017). Association between dietary energy density and incident type 2 diabetes in the women’s health initiative. J Acad Nutr Diet..

[28] Takeda Y, Fujihara K, Nedachi R, Ikeda I, Morikawa SY, Hatta M (2021). Comparing associations of dietary energy density and energy intake, macronutrients with obesity in patients with type 2 diabetes (JDDM 63). Nutrients..

[29] Hartman TJ, Gapstur SM, Gaudet MM, Shah R, Flanders WD, Wang Y (2016). Dietary energy density and postmenopausal breast cancer incidence in the cancer prevention study II nutrition cohort. J Nutr..

[30] Thomson CA, Crane TE, Garcia DO, Wertheim BC, Hingle M, Snetselaar L (2018). Association between dietary energy density and obesity-associated cancer: results from the Women’s Health Initiative. J Acad Nutr Diet..

[31] Zuraikat FM, St-Onge MP, Makarem N, Boege HL, Xi H, Aggarwal B (2021). Evening chronotype is associated with poorer habitual diet in US women, with dietary energy density mediating a relation of chronotype with cardiovascular health. J Nutr..

[32] Sebastian RS, Wilkinson Enns C, Goldman JD, Murayi T, Moshfegh AJ (2022). Late evening eating patterns among US adults vary in their associations with, and impact on, energy intake and diet quality: evidence from what we eat in america, national health and nutrition examination survey 2013-2016. J Acad Nutr Diet..

[33] Depner CM, Melanson EL, McHill AW, Wright KP (2018). Mistimed food intake and sleep alters 24-hour time-of-day patterns of the human plasma proteome. Proc Natl Acad Sci USA..

[34] Melendez-Fernandez OH, Liu JA, Nelson RJ (2023). Circadian rhythms disrupted by light at night and mistimed food intake alter hormonal rhythms and metabolism. Int J Mol Sci..

[35] Sato M, Nakamura K, Ogata H, Miyashita A, Nagasaka S, Omi N (2011). Acute effect of late evening meal on diurnal variation of blood glucose and energy metabolism. Obes Res Clin Pract..

[36] Bonham MP, Kaias E, Zimberg I, Leung GKW, Davis R, Sletten TL (2019). Effect of night time eating on postprandial triglyceride metabolism in healthy adults: a systematic literature review. J Biol Rhythms..

[37] Mirghani H (2021). The effect of breakfast skipping and late night eating on body mass index and glycemic control among patients with type 2 diabetes mellitus. Cureus..

[38] Tranah GJ, Blackwell T, Ancoli-Israel S, Paudel ML, Ensrud KE, Cauley JA (2010). Circadian activity rhythms and mortality: the study of osteoporotic fractures. J Am Geriatr Soc..

[39] Ruan W, Yuan X, Eltzschig HK (2021). Circadian rhythm as a therapeutic target. Nat Rev Drug Discov..

[40] Fang G, Chen Q, Li J, Lian X, Shi D (2024). The diurnal transcriptome reveals the reprogramming of lung adenocarcinoma cells under a time-restricted feeding-mimicking regimen. J Nutr.

[41] Fang G, Wang S, Chen Q, Luo H, Lian X, Shi D (2023). Time-restricted feeding affects the fecal microbiome metabolome and its diurnal oscillations in lung cancer mice. Neoplasia..

[42] Shi D, Fang G, Chen Q, Li J, Ruan X, Lian X (2023). Six-hour time-restricted feeding inhibits lung cancer progression and reshapes circadian metabolism. BMC Med.

[43] Huang Y, Guo H, Zhou Y, Guo J, Wang T, Zhao X (2020). The associations between fasting plasma glucose levels and mortality of COVID-19 in patients without diabetes. Diabetes Res Clin Pract..

[44] Rooney MR, Tang O, Pankow JS, Selvin E (2021). Glycaemic markers and all-cause mortality in older adults with and without diabetes: the Atherosclerosis Risk in Communities (ARIC) study. Diabetologia..

[45] Kim DK, Ko GJ, Choi YJ, Jeong KH, Moon JY, Lee SH (2022). Glycated hemoglobin levels and risk of all-cause and cause-specific mortality in hemodialysis patients with diabetes. Diabetes Res Clin Pract..

[46] Liu J, Zeng FF, Liu ZM, Zhang CX, Ling WH, Chen YM (2013). Effects of blood triglycerides on cardiovascular and all-cause mortality: a systematic review and meta-analysis of 61 prospective studies. Lipids Health Dis..

