# Supplementary material for: Night eating in timing, frequency, and food quality and risks of all-cause, cancer, and diabetes mortality: findings from national health and nutrition examination survey
Source: Nutr Diabetes. 2024 Feb 27;14:5. doi: 10.1038/s41387-024-00266-6 (PMC10899630; doi:10.1038/s41387-024-00266-6)
Supplement: Supplementary file 1 — Supplementary materials [file 41387_2024_266_MOESM1_ESM.docx]

**Supplementary material**

**
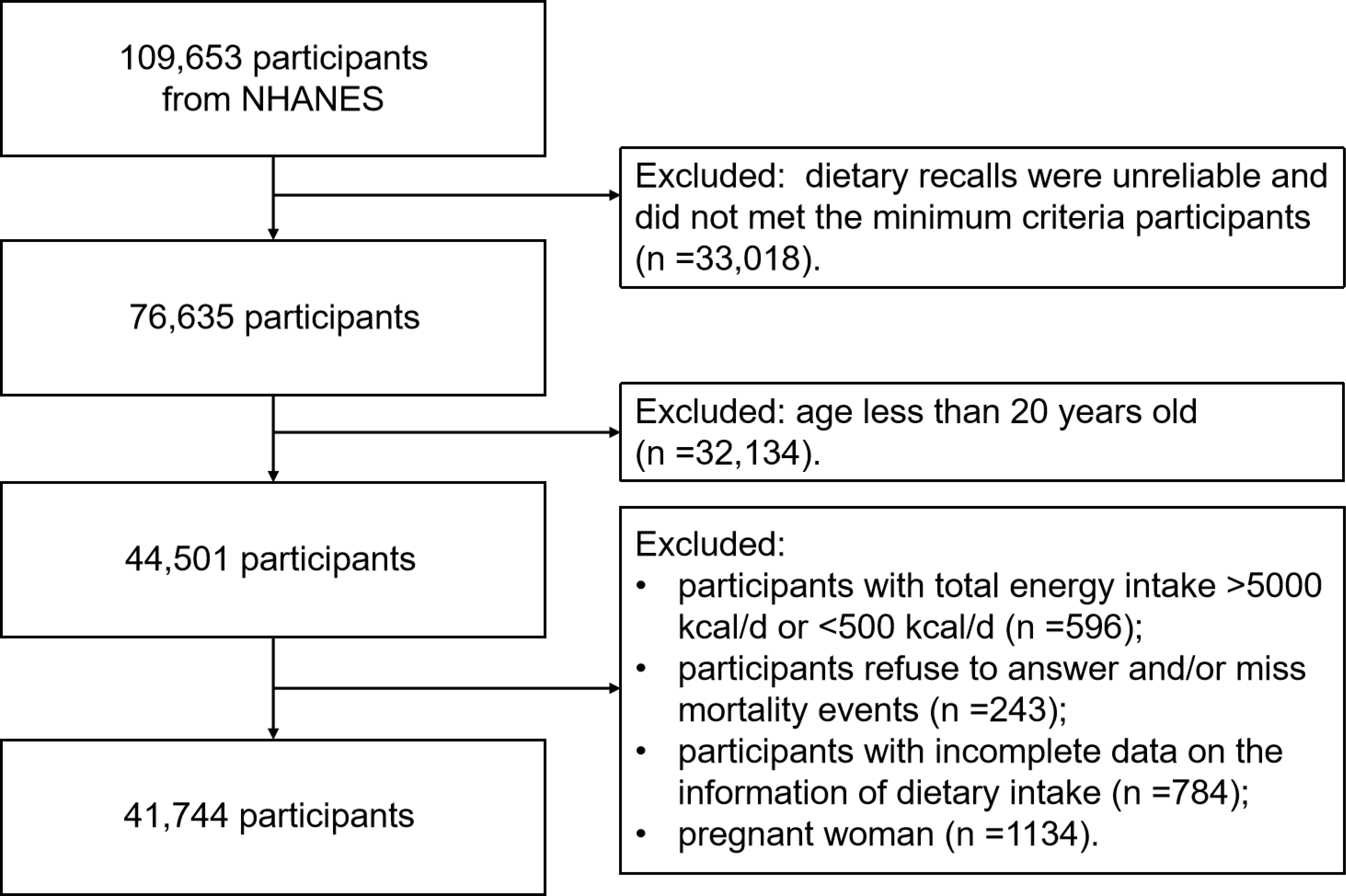
**

**Supplementary Fig. 1 Cohort flow diagram in NHANES 2002-2018.**


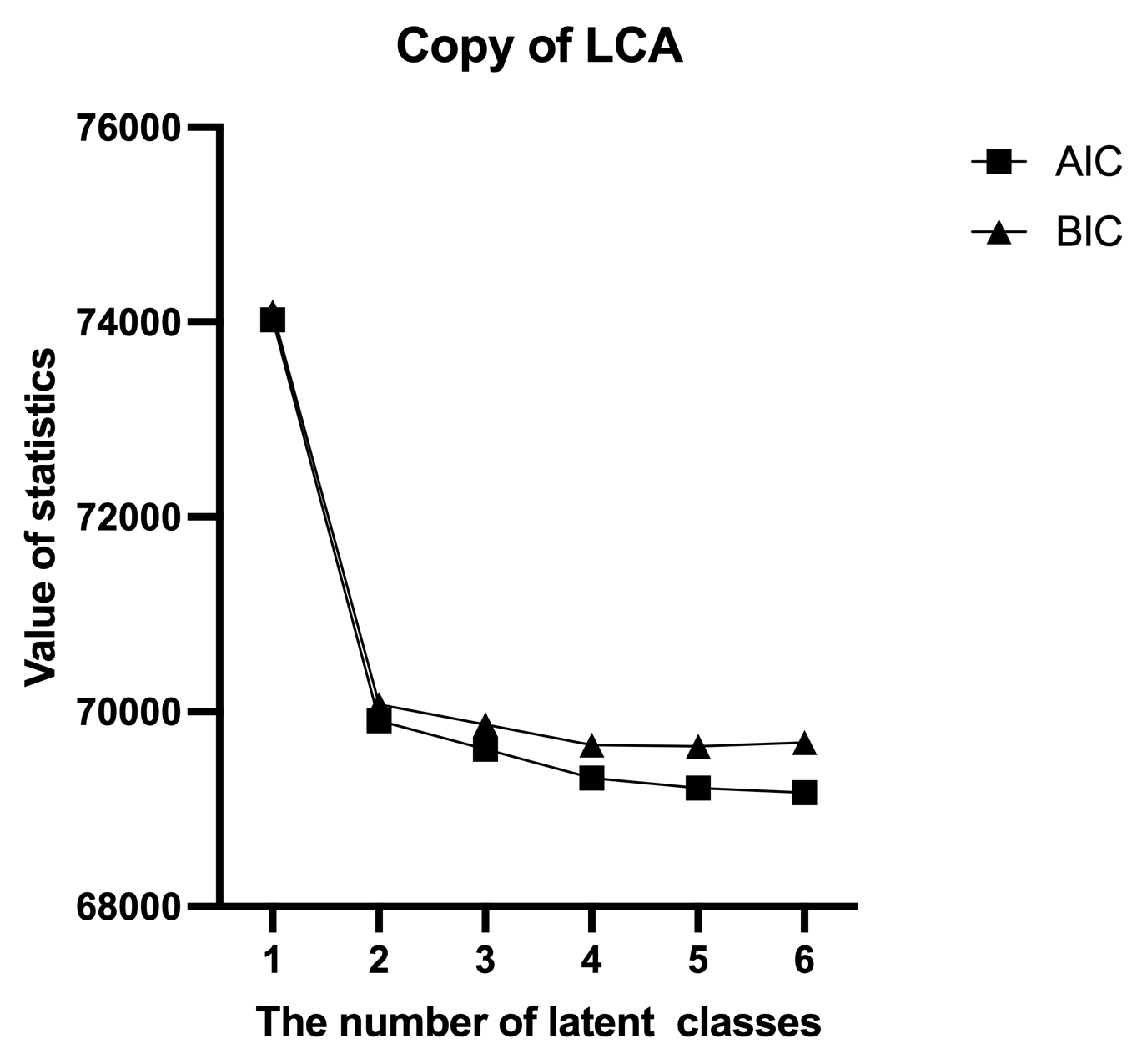


# Supplementary Fig. 2 AIC and BIC in models with different numbers of latent classes in the US NHANES.

Abbreviations: AIC, Akaike information criterion; BIC, Bayesian information criterion.

# Supplementary Table 1 Item-response probabilities in models with four latent classes in the NHANES

| **Item** | **Latent class 1** | **Latent class 2** | **Latent class 3** | **Latent class 4** |
| --- | --- | --- | --- | --- |
| Energy 1 | 0.87 | 0.35 | 0.20 | 0.06 |
| Energy 2 | 0.13 | 0.65 | 0.80 | 0.94 |
| Fruits 1 | 0.43 | 0.65 | 0.48 | 0.51 |
| Fruits 2 | 0.57 | 0.35 | 0.52 | 0.49 |
| Vegetables 1 | 0.79 | 0.90 | 0.43 | 0.43 |
| Vegetables 2 | 0.21 | 0.10 | 0.57 | 0.57 |
| Whole grians 1 | 0.83 | 0.58 | 0.31 | 0.41 |
| Whole grians 2 | 0.17 | 0.42 | 0.69 | 0.59 |
| Dairy 1 | 0.65 | 0.58 | 0.55 | 0.38 |
| Dairy 2 | 0.35 | 0.42 | 0.45 | 0.62 |
| Protein foods 1 | 0.94 | 0.89 | 0.37 | 0.33 |
| Protein foods 2 | 0.06 | 0.11 | 0.63 | 0.67 |
| Refined grains 1 | 0.96 | 0.81 | 0.45 | 0.23 |
| Refined grains 2 | 0.04 | 0.19 | 0.55 | 0.77 |
| Add sugars 1 | 0.89 | 0.21 | 0.57 | 0.36 |
| Add sugars 2 | 0.11 | 0.79 | 0.43 | 0.64 |
| Oils 1 | 0.88 | 0.85 | 0.28 | 0.37 |
| Oils 2 | 0.12 | 0.15 | 0.72 | 0.63 |
| Solid fats 1 | 0.95 | 0.43 | 1.00 | 0.00 |
| Solid fats 2 | 0.05 | 0.57 | 0.00 | 1.00 |
| Alcoholic drinking 1 | 0.95 | 0.32 | 0.43 | 0.41 |
| Alcoholic drinking 2 | 0.05 | 0.68 | 0.57 | 0.59 |

NA=not available. Energy intake 1 to 2 respectively referred to the evening energy intake <200 kcal and ≥200 kcal. For other items, category 1 to 2 respectively referred to the intake level of less than the median and greater than or equal to the median.

**Supplementary Table 2 Food intake in different latent class**

| **Food groups** | **Latent class 1**  **(VL-energy intake)** | **Latent class 2**  **(L-energy intake)** | **Latent class 3**  **(M-energy intake)** | **Latent class 4**  **(L-energy intake)** |
| --- | --- | --- | --- | --- |
| Energy intake (kcal) | 122.21± 2.49 | 342.91±12.14 | 518.80±12.06 | 723.06±12.19 |
| Low energy density foods intake (serving) |  |  |  |  |
| Fruits | 0.69±0.03 | 0.35±0.06 | 0.69±0.04 | 0.89±0.05 |
| Vegetables | 0.29±0.02 | 0.17±0.02 | 0.81±0.05 | 0.76±0.03 |
| Whole grains | 0.43±0.03 | 0.73±0.08 | 1.28±0.06 | 1.22±0.06 |
| Dairies | 0.42±0.02 | 0.42±0.03 | 0.54±0.03 | 0.96±0.03 |
| Protein foods | 0.28±0.04 | 0.20±0.04 | 2.40±0.11 | 2.78±0.08 |
| High energy density foods intake (serving) |  |  |  |  |
| Refined grains | 0.41±0.03 | 0.68±0.05 | 1.70±0.07 | 2.92±0.07 |
| Add sugars | 1.31±0.08 | 7.82±0.23 | 4.31±0.26 | 7.85±0.24 |
| Oils | 1.28±0.15 | 1.31±0.22 | 12.08±0.55 | 10.35±0.36 |
| Solid fats | 1.83±0.18 | 10.04±0.51 | 1.84±0.15 | 21.38±0.45 |
| Alcoholic drinkings | 0.35±0.10 | 2.55±0.28 | 1.89±0.19 | 2.61±0.14 |

**Supplementary Table 3 Missing information of covariates**

| **Charicteristics** | **Count** | **Percentage (%)** |
| --- | --- | --- |
| Age | 0 | 0 |
| Dietary energy intake | 0 | 0 |
| BMI | 659 | 1.58 |
| Physical acitivity | 2978 | 7.13 |
| Adherence to HEI-2015 scroe | 0 | 0 |
| Sex | 0 | 0 |
| Race | 0 | 0 |
| Education | 40 | 0.1 |
| Income | 3434 | 8.23 |
| Smoking status | 25 | 0.06 |
| Hypertension | 74 | 0.18 |
| CVD | 0 | 0 |
| T2D | 0 | 0 |
| Cancer | 0 | 0 |
| Hyperlipidemia | 0 | 0 |
| Dietary_supplements_taken | 2477 | 5.93 |
| Drinking status | 3273 | 7.84 |
| Sleep hours | 8442 | 20.22 |
| Glycohemoglobin | 1642 | 3.93 |
| Triglycerides | 2196 | 5.26 |
| Fasting glucose | 2163 | 5.18 |
| OGTT | 1474 | 3.53 |
| Total_cholesterol | 2182 | 5.23 |
| Fasting time | 566 | 1.36 |

# Supplementary Table 4 Adjusted HR (95% CI) of all-cause, diabetes, and cancer mortality with timing of night eating

| **Cause of mortality** | **Timing of night eating** | | | | | | | ***P* trend** |
| --- | --- | --- | --- | --- | --- | --- | --- | --- |
|  | **No night eating** | **22:00~23:00** | **23:00~00:00** | **00:00~1:00** | **1:00~2:00** | **2:00~3:00** | **3:00~4:00** |  |
| All-cause mortality |  |  |  |  |  |  |  |  |
| Case/N | 4910/31362 | 741/6262 | 244/2354 | 46/588 | 39/417 | 45/375 | 41/386 |  |
| Model 1 | 1.00 (Reference) | 1.07 (0.95, 1.19) | 1.38 (1.16, 1.65) | 1.44 (1.04, 1.98) | 1.60 (1.02, 2.52) | 1.30 (0.88, 1.94) | 1.35 (0.89, 2.04) | <0.001 |
| Model 2 | 1.00 (Reference) | 1.11 (0.99, 1.23) | 1.34 (1.11, 1.60) | 1.42 (1.04, 1.95) | 1.57 (1.01, 2.44) | 1.23 (0.82, 1.85) | 1.27 (0.84, 1.93) | <0.001 |
| Model 3 | 1.00 (Reference) | 1.04 (0.94, 1.16) | 1.29 (1.08, 1.54) | 1.38 (1.02, 1.88) | 1.49 (0.97, 2.30) | 1.12 (0.73, 1.71) | 1.22 (0.80, 1.86) | 0.002 |
| Sensitivity analysis ^a^ | 1.00 (Reference) | 1.04 (0.94, 1.16) | 1.28 (1.07, 1.53) | 1.39 (1.02, 1.89) | 1.49 (0.97, 2.30) | 1.12 (0.74, 1.71) | 1.22 (0.80, 1.86) | 0.002 |
| Cancer mortality |  |  |  |  |  |  |  |  |
| Case/N | 1103/31362 | 179/6262 | 52/2354 | 14/588 | 14/417 | 9/375 | 10/386 |  |
| Model 1 | 1.00 (Reference) | 1.06 (0.87, 1.31) | 1.03 (0.65, 1.63) | 1.51 (0.82, 2.79) | 2.08 (0.99, 4.35) | 1.09 (0.49, 2.42) | 1.05 (0.42, 2.66) | 0.181 |
| Model 2 | 1.00 (Reference) | 1.08 (0.88, 1.31) | 1.01 (0.64, 1.59) | 1.50 (0.83, 2.71) | 2.05 (0.98, 4.26) | 1.05 (0.47, 2.34) | 1.01 (0.40, 2.55) | 0.21 |
| Model 3 | 1.00 (Reference) | 1.05 (0.86, 1.29) | 0.95 (0.60, 1.50) | 1.55 (0.86, 2.82) | 2.09 (1.03, 4.25) | 0.92 (0.42, 2.03) | 0.89 (0.35, 2.23) | 0.417 |
| Sensitivity analysis ^a^ | 1.00 (Reference) | 1.06 (0.86, 1.30) | 0.94 (0.59, 1.49) | 1.56 (0.86, 2.83) | 2.10 (1.03, 4.26) | 0.93 (0.42, 2.03) | 0.88 (0.35, 2.23) | 0.421 |
| Diabetes mortality |  |  |  |  |  |  |  |  |
| Case/N | 162/31362 | 27/6262 | 14/2354 | 1/588 | 1/417 | 1/375 | 0/386 |  |
| Model 1 | 1.00 (Reference) | 1.96 (1.18, 3.24) | 2.68 (1.37, 5.25) | 0.47 (0.07, 3.34) | 1.10 (0.14, 8.63) | 0.48 (0.06, 3.62) | 0.00 (0.00, 0.00) | 0.016 |
| Model 2 | 1.00 (Reference) | 2.13 (1.31, 3.46) | 2.62 (1.32, 5.19) | 0.53 (0.07, 3.92) | 1.20 (0.15, 9.52) | 0.53 (0.07, 3.90) | 0.00 (0.00, 0.00) | 0.007 |
| Model 3 | 1.00 (Reference) | 1.68 (1.07, 2.62) | 2.31 (1.21, 4.40) | 0.60 (0.08, 4.65) | 1.13 (0.15, 8.69) | 0.59 (0.08, 4.45) | 0.00 (0.00, 0.00) | 0.026 |
| Sensitivity analysis ^a^ | 1.00 (Reference) | 1.67 (1.07, 2.61) | 2.32 (1.22, 4.42) | 0.60 (0.08, 4.61) | 1.12 (0.15, 8.68) | 0.59 (0.08, 4.43) | 0.00 (0.00, 0.00) | 0.027 |

Abbreviations: aHR, adjusted hazard ratio; CI, confidence intervals; Ref., reference; HEI-2015, Healthy Eating Index 2015; CVD, cardiovascular disease.

* aHR (95%CI) was estimated by weighted Cox regression analyses. Date is shown as aHR with 95%CI.

*Represented the significant association between timing of night eating and mortality.

Model 1 adjusted for age and sex.

Model 2 further adjusted for education, race/ethnicity, family income, and BMI.

Model 3 further adjusted for dietary energy intake, drinking status, smoking status, physical activity, diabetes, hypertension, hyperlipidemia, CVD, cancer, adherence to HEI-2015 score, and dietary supplement use.

^a^The sensitivity analysis further adjusted for sleep times.

# Supplementary Table 5 Adjusted HR (95% CI) of all-cause, diabetes, and cancer mortality with night eating frequency

| **Cause of mortality** | **Night eating frequency** | | | ***P* trend** |
| --- | --- | --- | --- | --- |
|  | **No night eating** | **One time** | **Two times or over** |  |
| All-cause mortality |  |  |  |  |
| Case/N | 4910/31362 | 1035/9050 | 121/1332 |  |
| Model 1 | 1.00 (Reference) | 1.14 (1.04, 1.25) | 1.46 (1.14, 1.85) | <0.001 |
| Model 2 | 1.00 (Reference) | 1.16 (1.06, 1.26) | 1.49 (1.18, 1.88) | <0.001 |
| Model 3 | 1.00 (Reference) | 1.10 (1.01, 1.20) | 1.38 (1.09, 1.75) | 0.002 |
| Sensitivity analysis ^a^ | 1.00 (Reference) | 1.10 (1.01, 1.20) | 1.37 (1.08, 1.74) | 0.002 |
| Cancer mortality |  |  |  |  |
| Case/N | 1103/31362 | 247/9050 | 31/1332 |  |
| Model 1 | 1.00 (Reference) | 1.10 (0.91, 1.32) | 1.18 (0.70, 1.99) | 0.26 |
| Model 2 | 1.00 (Reference) | 1.11 (0.92, 1.32) | 1.21 (0.72, 2.03) | 0.206 |
| Model 3 | 1.00 (Reference) | 1.06 (0.89, 1.28) | 1.10 (0.66, 1.85) | 0.451 |
| Sensitivity analysis ^a^ | 1.00 (Reference) | 1.07 (0.89, 1.28) | 1.08 (0.64, 1.81) | 0.471 |
| Diabetes mortality |  |  |  |  |
| Case/N | 162/31362 | 42/9050 | 2/1332 |  |
| Model 1 | 1.00 (Reference) | 1.97 (1.30, 3.00) | 0.95 (0.22, 4.03) | 0.007 |
| Model 2 | 1.00 (Reference) | 2.09 (1.40, 3.12) | 1.07 (0.25, 4.60) | 0.002 |
| Model 3 | 1.00 (Reference) | 1.72 (1.20, 2.48) | 1.08 (0.25, 4.73) | 0.018 |
| Sensitivity analysis ^a^ | 1.00 (Reference) | 1.72 (1.19, 2.47) | 1.09 (0.25, 4.80) | 0.018 |

Abbreviations: aHR, adjusted hazard ratio; CI, confidence intervals; Ref., reference; HEI-2015, Healthy Eating Index 2015; CVD, cardiovascular disease.

* aHR (95%CI) was estimated by weighted Cox regression analyses. Date is shown as aHR with 95%CI.

*Represented the significant association between timing of night eating and mortality.

Model 1 adjusted for age and sex.

Model 2 further adjusted for education, race/ethnicity, family income, and BMI.

Model 3 further adjusted for dietary energy intake, drinking status, smoking status, physical activity, diabetes, hypertension, hyperlipidemia, CVD, cancer, adherence to HEI-2015 score, and dietary supplement use.

^a^The sensitivity analysis further adjusted for sleep times.

# Supplementary Table 6 Adjusted HR (95% CI) of all-cause, diabetes, and cancer mortality with food quality of night eating

| **Cause of mortality** | **Food quality of night eating** | | | | | ***P* trend** |
| --- | --- | --- | --- | --- | --- | --- |
|  | **No night eating** | **VL-energy intake** | **L-energy intake** | **M-energy intake** | **H-energy intake** |  |
| All-cause mortality |  |  |  |  |  |  |
| Case/N | 4910/31362 | 436/3113 | 141/1218 | 182/1868 | 397/4183 |  |
| Model 1 | 1.00 (Reference) | 1.03 (0.91, 1.16) | 1.12 (0.87, 1.44) | 1.25 (1.01, 1.53) | 1.34 (1.17, 1.54) | <0.001 |
| Model 2 | 1.00 (Reference) | 1.09 (0.97, 1.23) | 1.11 (0.87, 1.41) | 1.29 (1.05, 1.59) | 1.28 (1.11, 1.47) | <0.001 |
| Model 3 | 1.00 (Reference) | 1.05 (0.93, 1.18) | 1.05 (0.83, 1.33) | 1.28 (1.03, 1.58) | 1.21 (1.06, 1.38) | 0.001 |
| Sensitivity analysis ^a^ | 1.00 (Reference) | 1.05 (0.93, 1.18) | 1.05 (0.83, 1.33) | 1.28 (1.04, 1.58) | 1.21 (1.06, 1.38) | 0.001 |
| Cancer mortality |  |  |  |  |  |  |
| Case/N | 1103/31362 | 89/3113 | 34/1218 | 47/1868 | 108/4183 |  |
| Model 1 | 1.00 (Reference) | 0.84 (0.65, 1.08) | 1.20 (0.76, 1.89) | 1.16 (0.82, 1.64) | 1.39 (1.06, 1.83) | 0.024 |
| Model 2 | 1.00 (Reference) | 0.87 (0.68, 1.12) | 1.17 (0.75, 1.84) | 1.18 (0.84, 1.65) | 1.33 (1.01, 1.75) | 0.04 |
| Model 3 | 1.00 (Reference) | 0.85 (0.66, 1.10) | 1.17 (0.75, 1.84) | 1.22 (0.87, 1.73) | 1.30 (0.99, 1.71) | 0.047 |
| Sensitivity analysis ^a^ | 1.00 (Reference) | 0.85 (0.66, 1.09) | 1.17 (0.75, 1.84) | 1.22 (0.87, 1.73) | 1.30 (0.99, 1.71) | 0.046 |
| Diabetes mortality |  |  |  |  |  |  |
| Case/N | 162/31362 | 17/3113 | 6/1218 | 5/1868 | 16/4183 |  |
| Model 1 | 1.00 (Reference) | 1.38 (0.76, 2.49) | 2.14 (0.55, 8.40) | 1.97 (0.65, 5.99) | 2.35 (1.27, 4.34) | 0.001 |
| Model 2 | 1.00 (Reference) | 1.56 (0.87, 2.82) | 2.19 (0.57, 8.42) | 2.30 (0.77, 6.85) | 2.26 (1.23, 4.15) | 0.001 |
| Model 3 | 1.00 (Reference) | 1.31 (0.75, 2.28) | 1.94 (0.57, 6.62) | 2.01 (0.69, 5.83) | 1.97 (1.13, 3.45) | 0.003 |
| Sensitivity analysis ^a^ | 1.00 (Reference) | 1.31 (0.75, 2.28) | 1.93 (0.57, 6.60) | 2.00 (0.69, 5.79) | 1.97 (1.13, 3.44) | 0.004 |

Abbreviations: aHR, adjusted hazard ratio; CI, confidence intervals; Ref., reference; HEI-2015, Healthy Eating Index 2015; CVD, cardiovascular disease.

* aHR (95%CI) was estimated by weighted Cox regression analyses. Date is shown as aHR with 95%CI.

*Represented the significant association between timing of night eating and mortality.

Model 1 adjusted for age and sex.

Model 2 further adjusted for education, race/ethnicity, family income, and BMI.

Model 3 further adjusted for dietary energy intake, drinking status, smoking status, physical activity, diabetes, hypertension, hyperlipidemia, CVD, cancer, adherence to HEI-2015 score, and dietary supplement use.

^a^The sensitivity analysis further adjusted for sleep times.

# Supplementary Table 7 subgroup analyses for the associations between timing of night eating and all-cause, diabetes and cancer mortality

| **Cause of mortality** | **Case/N** | **Timing of night eating** | | | | | | | ***P* trend** | ***P*  interaction** |
| --- | --- | --- | --- | --- | --- | --- | --- | --- | --- | --- |
|  |  | **No night**  **eating** | **22:00~23:00** | **23:00~00:00** | **00:00~1:00** | **1:00~2:00** | **2:00~3:00** | **3:00~4:00** |  |  |
| All-cause mortality |  |  |  |  |  |  |  |  |  |  |
| **Age (years)** |  |  |  |  |  |  |  |  |  |  |
| age<65 | 1733/31181 | 1(Ref.) | 0.94 (0.79, 1.11) | 1.10 (0.83, 1.47) | 1.05 (0.68, 1.61) | 1.13 (0.61, 2.08) | 0.98 (0.57, 1.70) | 1.29 (0.72, 2.31) | 0.562 | 0.526 |
| age≥65 | 4333/10563 | 1(Ref.) | 1.02 (0.90, 1.16) | 1.25 (1.02, 1.54) | 1.26 (0.68, 2.34) | 1.37 (0.74, 2.51) | 1.11 (0.63, 1.97) | 0.99 (0.60, 1.62) | 0.143 | 0.526 |
| **Sex** |  |  |  |  |  |  |  |  |  |  |
| Men | 3413/20602 | 1(Ref.) | 1.03 (0.89, 1.20) | 1.37 (1.11, 1.70) | 1.03 (0.67, 1.58) | 1.22 (0.73, 2.04) | 1.11 (0.71, 1.74) | 1.40 (0.83, 2.36) | 0.019 | 0.92 |
| Women | 2653/21142 | 1(Ref.) | 1.05 (0.92, 1.21) | 1.14 (0.86, 1.51) | 1.72 (1.07, 2.76) | 2.09 (1.15, 3.79) | 1.22 (0.59, 2.51) | 1.03 (0.53, 2.02) | 0.027 | 0.92 |
| **Body mass index (kg/m^2^)** |  |  |  |  |  |  |  |  |  |  |
| >25 | 1887/12161 | 1(Ref.) | 0.97 (0.83, 1.14) | 1.23 (0.87, 1.75) | 1.47 (0.79, 2.73) | 1.01 (0.47, 2.15) | 1.85 (1.02, 3.33) | 0.69 (0.23, 2.07) | 0.145 | 0.748 |
| 25-29 | 2140/14064 | 1(Ref.) | 1.06 (0.89, 1.27) | 1.44 (1.09, 1.90) | 1.48 (0.78, 2.80) | 1.23 (0.77, 1.98) | 1.27 (0.65, 2.47) | 1.98 (1.06, 3.72) | 0.003 | 0.748 |
| ≥30 | 2039/15519 | 1(Ref.) | 1.07 (0.89, 1.29) | 1.14 (0.84, 1.54) | 0.92 (0.36, 2.33) | 2.10 (1.13, 3.93) | 0.60 (0.29, 1.25) | 1.16 (0.61, 2.21) | 0.289 | 0.748 |
| **Smoking status** |  |  |  |  |  |  |  |  |  |  |
| Never smoker | 2390/22583 | 1(Ref.) | 1.08 (0.91, 1.28) | 1.21 (0.92, 1.60) | 2.02 (1.18, 3.46) | 1.49 (0.66, 3.36) | 1.77 (1.00, 3.14) | 1.65 (0.72, 3.79) | 0.001 | 0.499 |
| Past smoker | 2447/10476 | 1(Ref.) | 1.01 (0.85, 1.20) | 1.24 (0.97, 1.59) | 1.28 (0.65, 2.51) | 1.25 (0.65, 2.40) | 1.08 (0.53, 2.20) | 1.53 (0.88, 2.66) | 0.103 | 0.499 |
| Current smoker | 1229/8685 | 1(Ref.) | 1.02 (0.84, 1.24) | 1.26 (0.89, 1.77) | 0.87 (0.41, 1.83) | 1.53 (0.85, 2.75) | 0.66 (0.32, 1.37) | 0.65 (0.27, 1.57) | 0.95 | 0.499 |
| **Drinking status** |  |  |  |  |  |  |  |  |  |  |
| Never drinker | 1053/6015 | 1(Ref.) | 1.14 (0.89, 1.46) | 0.84 (0.52, 1.37) | 2.31 (0.75, 7.11) | 2.42 (0.95, 6.17) | 0.92 (0.31, 2.68) | 0.57 (0.26, 1.22) | 0.167 | 0.71 |
| Past drinker | 2085/7439 | 1(Ref.) | 0.93 (0.79, 1.10) | 1.43 (1.10, 1.85) | 1.66 (0.81, 3.41) | 0.91 (0.38, 2.19) | 1.28 (0.64, 2.58) | 1.10 (0.52, 2.32) | 0.103 | 0.71 |
| Current drinker | 2928/28290 | 1(Ref.) | 1.09 (0.95, 1.25) | 1.28 (1.01, 1.63) | 1.04 (0.66, 1.64) | 1.49 (0.84, 2.64) | 1.17 (0.76, 1.80) | 1.43 (0.86, 2.38) | 0.002 | 0.71 |
| **HEI-2015 score** |  |  |  |  |  |  |  |  |  |  |
| <70 | 586/3743 | 1(Ref.) | 1.07 (0.72, 1.58) | 1.76 (1.02, 3.01) | 1.27 (0.46, 3.55) | 2.90 (1.22, 6.94) | 1.93 (0.86, 4.37) | 0.44 (0.06, 3.09) | 0.045 | 0.589 |
| ≥70 | 5480/38001 | 1(Ref.) | 1.04 (0.93, 1.16) | 1.25 (1.04, 1.49) | 1.40 (1.00, 1.95) | 1.44 (0.89, 2.33) | 1.11 (0.72, 1.72) | 1.36 (0.88, 2.08) | 0.003 | 0.589 |
| **Sleep hours** |  |  |  |  |  |  |  |  |  |  |
| <6 hours | 901/6189 | 1(Ref.) | 0.96 (0.75, 1.22) | 1.39 (0.90, 2.14) | 1.23 (0.59, 2.55) | 1.02 (0.34, 3.00) | 1.08 (0.51, 2.32) | 2.58 (1.24, 5.37) | 0.03 | 0.249 |
| ≥6 hours | 5165/35555 | 1(Ref.) | 1.05 (0.94, 1.19) | 1.24 (1.02, 1.51) | 1.43 (1.01, 2.01) | 1.64 (1.01, 2.66) | 1.09 (0.65, 1.85) | 0.85 (0.52, 1.40) | 0.025 | 0.249 |
|  |  |  |  |  |  |  |  |  |  |  |
| Cancer mortality |  |  |  |  |  |  |  |  |  |  |
| **Age (years)** |  |  |  |  |  |  |  |  |  |  |
| age<65 | 502/31181 | 1(Ref.) | 1.01 (0.73, 1.38) | 0.95 (0.53, 1.69) | 1.08 (0.50, 2.33) | 1.62 (0.66, 3.99) | 0.70 (0.24, 2.07) | 0.99 (0.26, 3.75) | 0.89 | 0.781 |
| age≥65 | 879/10563 | 1(Ref.) | 0.96 (0.75, 1.24) | 0.73 (0.40, 1.34) | 1.93 (0.75, 4.97) | 1.78 (0.58, 5.49) | 1.28 (0.44, 3.75) | 0.74 (0.27, 2.05) | 0.933 | 0.781 |
| **Sex** |  |  |  |  |  |  |  |  |  |  |
| Men | 829/20602 | 1(Ref.) | 1.16 (0.86, 1.56) | 0.90 (0.57, 1.42) | 1.84 (0.95, 3.59) | 2.06 (0.79, 5.35) | 0.70 (0.23, 2.12) | 1.35 (0.48, 3.80) | 0.242 | 0.88 |
| Women | 552/21142 | 1(Ref.) | 0.95 (0.71, 1.27) | 1.07 (0.47, 2.40) | 1.23 (0.38, 4.02) | 2.26 (0.84, 6.08) | 1.51 (0.53, 4.31) | 0.30 (0.04, 2.23) | 0.791 | 0.88 |
| **Body mass index (kg/m^2^)** |  |  |  |  |  |  |  |  |  |  |
| >25 | 397/12161 | 1(Ref.) | 1.15 (0.83, 1.59) | 1.20 (0.55, 2.61) | 1.55 (0.63, 3.82) | 1.54 (0.44, 5.36) | 0.78 (0.14, 4.47) | 0.35 (0.06, 1.90) | 0.559 | 0.675 |
| 25-29 | 495/14064 | 1(Ref.) | 0.98 (0.69, 1.39) | 0.82 (0.42, 1.58) | 1.60 (0.61, 4.22) | 1.55 (0.65, 3.72) | 1.38 (0.52, 3.68) | 2.04 (0.59, 7.07) | 0.364 | 0.675 |
| ≥30 | 489/15519 | 1(Ref.) | 1.05 (0.72, 1.53) | 0.86 (0.39, 1.88) | 1.43 (0.37, 5.59) | 2.93 (0.91, 9.42) | 0.97 (0.25, 3.73) | 0.45 (0.13, 1.58) | 0.815 | 0.675 |
| **Smoking status** |  |  |  |  |  |  |  |  |  |  |
| Never smoker | 456/22583 | 1(Ref.) | 1.18 (0.80, 1.75) | 1.52 (0.74, 3.13) | 2.69 (0.94, 7.69) | 0.44 (0.06, 3.42) | 0.00 (0.00, 0.00) | 2.18(0.35, 13.66) | 0.185 | 0.737 |
| Past smoker | 581/10476 | 1(Ref.) | 0.83 (0.61, 1.14) | 0.49 (0.26, 0.90) | 1.60 (0.54, 4.67) | 2.58 (0.98, 6.82) | 1.19 (0.45, 3.19) | 0.83 (0.24, 2.87) | 0.532 | 0.737 |
| Current smoker | 344/8685 | 1(Ref.) | 1.22 (0.83, 1.78) | 1.02 (0.49, 2.09) | 0.99 (0.47, 2.08) | 2.74 (0.98, 7.69) | 1.12 (0.36, 3.50) | 0.39 (0.13, 1.16) | 0.469 | 0.737 |
| **Drinking status** |  |  |  |  |  |  |  |  |  |  |
| Never drinker | 181/6015 | 1(Ref.) | 1.52 (0.83, 2.78) | 0.90 (0.28, 2.89) | 4.16 (0.69, 25.31) | 5.53 (1.68, 18.25) | 1.28 (0.22, 7.57) | 1.08 (0.13, 8.71) | 0.015 | 0.071 |
| Past drinker | 477/7439 | 1(Ref.) | 0.99 (0.67, 1.45) | 1.18 (0.51, 2.73) | 1.68 (0.57, 4.90) | 0.34 (0.05, 2.62) | 1.26 (0.35, 4.51) | 0.39 (0.06, 2.51) | 0.915 | 0.071 |
| Current drinker | 723/28290 | 1(Ref.) | 1.01 (0.78, 1.32) | 0.84 (0.48, 1.45) | 1.32 (0.63, 2.77) | 1.99 (0.78, 5.06) | 0.78 (0.23, 2.67) | 1.09 (0.38, 3.12) | 0.726 | 0.071 |
| **HEI-2015 score** |  |  |  |  |  |  |  |  |  |  |
| <70 | 129/3743 | 1(Ref.) | 1.39 (0.65, 2.98) | 0.40 (0.11, 1.39) | 1.47 (0.16, 13.30) | 4.73 (0.70, 32.11) | 1.76 (0.26, 11.93) | 0.00 (0.00, 0.00) | 0.49 | 0.943 |
| ≥70 | 1252/38001 | 1(Ref.) | 1.04 (0.83, 1.31) | 1.03 (0.65, 1.64) | 1.61 (0.86, 3.02) | 2.01 (0.92, 4.38) | 0.95 (0.40, 2.23) | 1.05 (0.42, 2.65) | 0.296 | 0.943 |
| **Sleep hours** |  |  |  |  |  |  |  |  |  |  |
| <6 hours | 211/6189 | 1(Ref.) | 1.09 (0.67, 1.78) | 1.95 (0.88, 4.33) | 1.52 (0.33, 6.95) | 1.37 (0.27, 6.98) | 1.51 (0.38, 5.92) | 2.79 (0.73, 10.59) | 0.048 | 0.085 |
| ≥6 hours | 1170/35555 | 1(Ref.) | 1.05 (0.83, 1.32) | 0.75 (0.45, 1.26) | 1.55 (0.79, 3.02) | 2.26 (1.04, 4.93) | 0.79 (0.30, 2.10) | 0.36 (0.15, 0.84) | 0.797 | 0.085 |
|  |  |  |  |  |  |  |  |  |  |  |
| Diabetes mortality |  |  |  |  |  |  |  |  |  |  |
| **Age (years)** |  |  |  |  |  |  |  |  |  |  |
| age<65 | 87/31181 | 1(Ref.) | 1.79 (0.94, 3.43) | 1.98 (0.80, 4.87) | 0.00 (0.00, 0.00) | 0.00 (0.00, 0.00) | 0.00 (0.00, 0.00) | 0.00 (0.00, 0.00) | 0.379 | 0.286 |
| age≥65 | 119/10563 | 1(Ref.) | 1.46 (0.69, 3.08) | 3.07 (1.46, 6.44) | 2.54 (0.30, 21.24) | 6.51 (0.98, 43.19) | 1.40 (0.18, 11.10) | 0.00 (0.00, 0.00) | 0.014 | 0.286 |
| **Sex** |  |  |  |  |  |  |  |  |  |  |
| Men | 104/20602 | 1(Ref.) | 2.04 (1.06, 3.89) | 2.87 (1.17, 7.05) | 0.00 (0.00, 0.00) | 0.00 (0.00, 0.00) | 1.06 (0.15, 7.68) | 0.00 (0.00, 0.00) | 0.081 | 0.666 |
| Women | 102/21142 | 1(Ref.) | 1.26 (0.61, 2.61) | 1.84 (0.76, 4.43) | 1.39 (0.19, 10.13) | 3.70 (0.52, 26.49) | 0.00 (0.00, 0.00) | 0.00 (0.00, 0.00) | 0.31 | 0.666 |
| **Body mass index (kg/m^2^)** |  |  |  |  |  |  |  |  |  |  |
| >25 | 45/12161 | 1(Ref.) | 0.84 (0.26, 2.66) | 0.43 (0.10, 1.97) | 0.00 (0.00, 0.00) | 0.00 (0.00, 0.00) | 0.00 (0.00, 0.00) | 0.00 (0.00, 0.00) | 0.157 | 0.586 |
| 25-29 | 62/14064 | 1(Ref.) | 2.42 (1.11, 5.31) | 5.89 (2.41, 14.38) | 0.00 (0.00, 0.00) | 0.00 (0.00, 0.00) | 0.00 (0.00, 0.00) | 0.00 (0.00, 0.00) | 0.003 | 0.586 |
| ≥30 | 99/15519 | 1(Ref.) | 1.57 (0.80, 3.09) | 1.63 (0.62, 4.26) | 1.98 (0.24, 16.05) | 2.96 (0.38, 22.98) | 1.04 (0.12, 8.65) | 0.00 (0.00, 0.00) | 0.119 | 0.586 |
| **Smoking status** |  |  |  |  |  |  |  |  |  |  |
| Never smoker | 99/22583 | 1(Ref.) | 1.55 (0.83, 2.89) | 2.65 (1.07, 6.52) | 1.66 (0.20, 13.88) | 0.00 (0.00, 0.00) | 0.00 (0.00, 0.00) | 0.00 (0.00, 0.00) | 0.108 | 0.619 |
| Past smoker | 75/10476 | 1(Ref.) | 1.77 (0.72, 4.35) | 2.49 (0.91, 6.81) | 0.00 (0.00, 0.00) | 9.19 (1.08, 77.89) | 1.65 (0.23, 11.64) | 0.00 (0.00, 0.00) | 0.028 | 0.619 |
| Current smoker | 32/8685 | 1(Ref.) | 0.95 (0.38, 2.36) | 1.11 (0.25, 5.04) | 0.00 (0.00, 0.00) | 0.00 (0.00, 0.00) | 0.00 (0.00, 0.00) | 0.00 (0.00, 0.00) | 0.309 | 0.619 |
| **Drinking status** |  |  |  |  |  |  |  |  |  |  |
| Never drinker | 63/6015 | 1(Ref.) | 1.71 (0.80, 3.64) | 1.73 (0.54, 5.48) | 3.61 (0.35, 36.99) | 0.00 (0.00, 0.00) | 0.00 (0.00, 0.00) | 0.00 (0.00, 0.00) | 0.147 | 0.331 |
| Past drinker | 69/7439 | 1(Ref.) | 1.89 (0.86, 4.12) | 2.89 (1.03, 8.06) | 0.00 (0.00, 0.00) | 7.29 (0.99, 53.57) | 1.56 (0.19, 13.01) | 0.00 (0.00, 0.00) | 0.028 | 0.331 |
| Current drinker | 74/28290 | 1(Ref.) | 1.55 (0.63, 3.80) | 1.99 (0.88, 4.49) | 0.00 (0.00, 0.00) | 0.00 (0.00, 0.00) | 0.00 (0.00, 0.00) | 0.00 (0.00, 0.00) | 0.682 | 0.331 |
| **HEI-2015 score** |  |  |  |  |  |  |  |  |  |  |
| <70 | 21/3743 | 1(Ref.) | 0.25 (0.03, 2.44) | 1.81 (0.68, 4.81) | 0.00 (0.00, 0.00) | 0.00 (0.00, 0.00) | 0.00 (0.00, 0.00) | 0.00 (0.00, 0.00) | 0.301 | 0.771 |
| ≥70 | 185/38001 | 1(Ref.) | 1.79 (1.12, 2.85) | 2.10 (1.02, 4.33) | 0.63 (0.08, 4.71) | 1.22 (0.16, 9.53) | 0.62 (0.08, 4.62) | 0.00 (0.00, 0.00) | 0.043 | 0.771 |
| **Sleep hours** |  |  |  |  |  |  |  |  |  |  |
| <6 hours | 40/6189 | 1(Ref.) | 0.23 (0.05, 1.01) | 3.29 (1.31, 8.22) | 0.00 (0.00, 0.00) | 0.00 (0.00, 0.00) | 0.00 (0.00, 0.00) | 0.00 (0.00, 0.00) | 0.876 | 0.443 |
| ≥6 hours | 166/35555 | 1(Ref.) | 2.10 (1.32, 3.33) | 1.88 (0.74, 4.80) | 0.74 (0.09, 5.87) | 1.36 (0.17, 10.89) | 0.95 (0.12, 7.21) | 0.00 (0.00, 0.00) | 0.021 | 0.443 |

Abbreviations: aHR, adjusted hazard ratio; CI, confidence intervals; Ref., reference; HEI-2015, Healthy Eating Index 2015; CVD, cardiovascular disease.

* aHR (95%CI) was estimated by weighted Cox regression analyses. Date is shown as aHR with 95%CI.

Model adjusted for age, sex, education, race/ethnicity, family income, BMI, dietary energy intake, drinking status, smoking status, physical activity, diabetes, hypertension, hyperlipidemia, CVD, cancer, adherence to HEI-2015 score, and dietary supplement use, except for subgroup variables.

# Supplementary Table 8 subgroup analyses for the association between night eating frequency and all-cause, cancer and diabetes mortality

| **Cause of mortality** | **Case/N** | **Night eating frequency** | | | ***P* trend** | ***P*  interaction** |
| --- | --- | --- | --- | --- | --- | --- |
|  |  | **No night eating** | **One time** | **Two times or over** |  |  |
| All-cause mortality |  |  |  |  |  |  |
| **Age (years)** |  |  |  |  |  |  |
| age<65 | 1733/31181 | 1.00 (Reference) | 0.99 (0.87, 1.14) | 1.04 (0.72, 1.52) | 0.92 | 0.235 |
| age≥65 | 4333/10563 | 1.00 (Reference) | 1.05 (0.93, 1.18) | 1.47 (1.12, 1.92) | 0.071 | 0.235 |
| **Sex** |  |  |  |  |  |  |
| Men | 3413/20602 | 1.00 (Reference) | 1.11 (0.98, 1.25) | 1.28 (0.93, 1.75) | 0.037 | 0.938 |
| Women | 2653/21142 | 1.00 (Reference) | 1.08 (0.95, 1.22) | 1.64 (1.17, 2.30) | 0.013 | 0.938 |
| **Body mass index (kg/m^2^)** |  |  |  |  |  |  |
| >25 | 1887/12161 | 1.00 (Reference) | 1.06 (0.90, 1.23) | 1.27 (0.81, 1.99) | 0.246 | 0.997 |
| 25-29 | 2140/14064 | 1.00 (Reference) | 1.14 (0.98, 1.32) | 1.88 (1.30, 2.73) | 0.004 | 0.997 |
| ≥30 | 2039/15519 | 1.00 (Reference) | 1.08 (0.93, 1.26) | 1.14 (0.70, 1.87) | 0.276 | 0.997 |
| **Smoking status** |  |  |  |  |  |  |
| Never smoker | 1229/8685 | 1.00 (Reference) | 1.06 (0.88, 1.26) | 1.00 (0.65, 1.56) | 0.627 | 0.329 |
| Past smoker | 2390/22583 | 1.00 (Reference) | 1.16 (1.01, 1.34) | 1.42 (0.89, 2.28) | 0.016 | 0.329 |
| Current smoker | 2447/10476 | 1.00 (Reference) | 1.03 (0.89, 1.20) | 1.65 (1.19, 2.28) | 0.064 | 0.329 |
| **Drinking status** |  |  |  |  |  |  |
| Never drinker | 1053/6015 | 1.00 (Reference) | 1.19 (0.96, 1.48) | 0.76 (0.35, 1.65) | 0.296 | 0.344 |
| Past drinker | 2085/7439 | 1.00 (Reference) | 1.06 (0.92, 1.22) | 1.24 (0.87, 1.77) | 0.198 | 0.344 |
| Current drinker | 2928/28290 | 1.00 (Reference) | 1.11 (1.00, 1.22) | 1.62 (1.21, 2.17) | 0.001 | 0.344 |
| **HEI-2015 score** |  |  |  |  |  |  |
| <70 | 502/31181 | 1.00 (Reference) | 1.04 (0.79, 1.36) | 0.83 (0.39, 1.76) | 0.895 | 0.626 |
| ≥70 | 879/10563 | 1.00 (Reference) | 0.94 (0.73, 1.19) | 1.28 (0.76, 2.18) | 0.918 | 0.626 |
| **Sleep hours** |  |  |  |  |  |  |
| <6 hours | 901/6189 | 1.00 (Reference) | 1.08 (0.88, 1.33) | 1.54 (0.97, 2.43) | 0.095 | 0.642 |
| ≥6 hours | 5165/35555 | 1.00 (Reference) | 1.09 (0.99, 1.20) | 1.34 (1.02, 1.76) | 0.011 | 0.642 |
|  |  |  |  |  |  |  |
| Cancer mortality |  |  |  |  |  |  |
| **Age (years)** |  |  |  |  |  |  |
| age<65 | 502/31181 | 1.00 (Reference) | 0.99 (0.75, 1.30) | 0.74 (0.34, 1.58) | 0.543 | 0.526 |
| age≥65 | 879/10563 | 1.00 (Reference) | 0.93 (0.73, 1.20) | 1.24 (0.72, 2.13) | 0.874 | 0.526 |
| **Sex** |  |  |  |  |  |  |
| Men | 829/20602 | 1.00 (Reference) | 1.17 (0.90, 1.52) | 1.00 (0.58, 1.71) | 0.315 | 0.971 |
| Women | 552/21142 | 1.00 (Reference) | 0.97 (0.73, 1.29) | 1.48 (0.61, 3.59) | 0.743 | 0.971 |
| **Body mass index (kg/m^2^)** |  |  |  |  |  |  |
| >25 | 397/12161 | 1.00 (Reference) | 1.18 (0.86, 1.62) | 0.94 (0.47, 1.89) | 0.464 | 0.733 |
| 25-29 | 495/14064 | 1.00 (Reference) | 1.01 (0.74, 1.37) | 1.38 (0.73, 2.59) | 0.647 | 0.733 |
| ≥30 | 489/15519 | 1.00 (Reference) | 1.03 (0.75, 1.43) | 1.14 (0.36, 3.69) | 0.774 | 0.733 |
| **Smoking status** |  |  |  |  |  |  |
| Never smoker | 456/22583 | 1.00 (Reference) | 1.27 (0.90, 1.80) | 1.22 (0.25, 5.92) | 0.195 | 0.84 |
| Past smoker | 581/10476 | 1.00 (Reference) | 0.82 (0.62, 1.09) | 1.11 (0.58, 2.13) | 0.29 | 0.84 |
| Current smoker | 344/8685 | 1.00 (Reference) | 1.20 (0.86, 1.66) | 1.02 (0.53, 1.95) | 0.392 | 0.84 |
| **Drinking status** |  |  |  |  |  |  |
| Never drinker | 181/6015 | 1.00 (Reference) | 1.65 (0.98, 2.78) | 1.70 (0.58, 4.97) | 0.043 | 0.072 |
| Past drinker | 477/7439 | 1.00 (Reference) | 1.07 (0.75, 1.52) | 0.77 (0.30, 1.98) | 0.953 | 0.072 |
| Current drinker | 723/28290 | 1.00 (Reference) | 1.01 (0.80, 1.27) | 1.31 (0.67, 2.57) | 0.618 | 0.072 |
| **HEI-2015 score** |  |  |  |  |  |  |
| <70 | 129/3743 | 1.00 (Reference) | 1.17 (0.58, 2.35) | 2.49 (0.75, 8.32) | 0.327 | 0.645 |
| ≥70 | 1252/38001 | 1.00 (Reference) | 1.08 (0.88, 1.32) | 1.10 (0.62, 1.93) | 0.429 | 0.645 |
| **Sleep hours** |  |  |  |  |  |  |
| <6 hours | 211/6189 | 1.00 (Reference) | 1.43 (0.94, 2.15) | 1.16 (0.47, 2.88) | 0.122 | 0.253 |
| ≥6 hours | 1170/35555 | 1.00 (Reference) | 1.00 (0.81, 1.22) | 1.09 (0.60, 2.00) | 0.887 | 0.253 |
|  |  |  |  |  |  |  |
| Diabetes mortality |  |  |  |  |  |  |
| **Age (years)** |  |  |  |  |  |  |
| age<65 | 104/20602 | 1.00 (Reference) | 2.04 (1.12, 3.72) | 1.03 (0.12, 8.69) | 0.047 | 0.505 |
| age≥65 | 102/21142 | 1.00 (Reference) | 1.37 (0.74, 2.54) | 1.43 (0.19, 10.49) | 0.329 | 0.505 |
| **Sex** |  |  |  |  |  |  |
| Men | 104/20602 | 1.00 (Reference) | 2.04 (1.14, 3.64) | 0.93 (0.10, 8.67) | 0.054 | 0.485 |
| Women | 102/21142 | 1.00 (Reference) | 1.38 (0.75, 2.54) | 1.39 (0.19, 10.25) | 0.31 | 0.485 |
| **Body mass index (kg/m^2^)** |  |  |  |  |  |  |
| >25 | 45/12161 | 1.00 (Reference) | 0.75 (0.25, 2.26) | 0.00 (0.00, 0.00) | 0.224 | 0.531 |
| 25-29 | 62/14064 | 1.00 (Reference) | 2.81 (1.44, 5.47) | 3.25 (0.45, 23.23) | 0.001 | 0.531 |
| ≥30 | 99/15519 | 1.00 (Reference) | 1.59 (0.90, 2.80) | 1.17 (0.13, 10.15) | 0.159 | 0.531 |
| **Smoking status** |  |  |  |  |  |  |
| Never smoker | 99/22583 | 1.00 (Reference) | 1.59 (0.94, 2.71) | 2.22 (0.27, 18.25) | 0.069 | 0.688 |
| Past smoker | 75/10476 | 1.00 (Reference) | 2.13 (1.07, 4.26) | 0.00 (0.00, 0.00) | 0.107 | 0.688 |
| Current smoker | 32/8685 | 1.00 (Reference) | 0.78 (0.34, 1.79) | 1.01 (0.11, 9.53) | 0.745 | 0.688 |
| **Drinking status** |  |  |  |  |  |  |
| Never drinker | 63/6015 | 1.00 (Reference) | 1.79 (0.90, 3.59) | 0.00 (0.00, 0.00) | 0.188 | 0.605 |
| Past drinker | 69/7439 | 1.00 (Reference) | 2.02 (1.05, 3.90) | 2.27 (0.30, 17.22) | 0.025 | 0.605 |
| Current drinker | 74/28290 | 1.00 (Reference) | 1.52 (0.71, 3.23) | 0.85 (0.09, 7.76) | 0.466 | 0.605 |
| **HEI-2015 score** |  |  |  |  |  |  |
| <70 | 21/3743 | 1.00 (Reference) | 0.65 (0.30, 1.38) | 0.00 (0.00, 0.00) | 0.082 | 0.695 |
| ≥70 | 185/38001 | 1.00 (Reference) | 1.76 (1.18, 2.64) | 1.15 (0.26, 5.03) | 0.021 | 0.695 |
| **Sleep hours** |  |  |  |  |  |  |
| <6 hours | 40/6189 | 1.00 (Reference) | 0.78 (0.35, 1.71) | 1.40 (0.16, 12.27) | 0.891 | 0.319 |
| ≥6 hours | 166/35555 | 1.00 (Reference) | 1.99 (1.30, 3.03) | 0.82 (0.10, 6.67) | 0.011 | 0.319 |

Abbreviations: aHR, adjusted hazard ratio; CI, confidence intervals; Ref., reference; HEI-2015, Healthy Eating Index 2015; CVD, cardiovascular disease.

* aHR (95%CI) was estimated by weighted Cox regression analyses. Date is shown as aHR with 95%CI.

Model adjusted for age, sex, education, race/ethnicity, family income, BMI, dietary energy intake, drinking status, smoking status, physical activity, diabetes, hypertension, hyperlipidemia, CVD, cancer, adherence to HEI-2015 score, and dietary supplement use, except for subgroup variables..

# Supplementary Table 9 subgroup analyses for the association between food quality of night eating and all-cause, cancer, and diabetes mortality

| **Cause of mortality** | **Case/N** | **Food quality** | | | | | ***P* trend** | ***P* interaction** |
| --- | --- | --- | --- | --- | --- | --- | --- | --- |
|  |  | **No night eating** | **VL-energy intake** | **L-energy intake** | **M-energy intake** | **H-energy intake** |  |  |
| All-cause mortality |  |  |  |  |  |  |  |  |
| **Age (years)** |  |  |  |  |  |  |  |  |
| age<65 | 1733/31181 | 1.00 (Reference) | 0.99 (0.78, 1.26) | 0.77 (0.52, 1.13) | 1.15 (0.83, 1.59) | 1.02 (0.83, 1.24) | 0.805 | 0.399 |
| age≥65 | 4333/10563 | 1.00 (Reference) | 1.03 (0.90, 1.19) | 1.12 (0.78, 1.61) | 1.21 (0.93, 1.56) | 1.11 (0.94, 1.32) | 0.09 | 0.399 |
| **Sex** |  |  |  |  |  |  |  |  |
| Men | 3413/20602 | 1.00 (Reference) | 0.97 (0.81, 1.16) | 1.14 (0.78, 1.65) | 1.32 (1.00, 1.74) | 1.23 (1.03, 1.46) | 0.004 | 0.532 |
| Women | 2653/21142 | 1.00 (Reference) | 1.08 (0.92, 1.28) | 0.98 (0.76, 1.28) | 1.23 (0.95, 1.60) | 1.21 (0.98, 1.48) | 0.04 | 0.532 |
| **Body mass index (kg/m^2^)** |  |  |  |  |  |  |  |  |
| >25 | 1887/12161 | 1.00 (Reference) | 0.98 (0.79, 1.21) | 1.15 (0.79, 1.68) | 1.24 (0.85, 1.82) | 1.21 (0.93, 1.56) | 0.074 | 0.878 |
| 25-29 | 2140/14064 | 1.00 (Reference) | 1.07 (0.86, 1.34) | 0.95 (0.62, 1.48) | 1.50 (1.11, 2.03) | 1.29 (1.00, 1.67) | 0.008 | 0.878 |
| ≥30 | 2039/15519 | 1.00 (Reference) | 1.01 (0.82, 1.25) | 1.10 (0.78, 1.57) | 1.13 (0.77, 1.66) | 1.17 (0.94, 1.46) | 0.122 | 0.878 |
| **Smoking status** |  |  |  |  |  |  |  |  |
| Never smoker | 2390/22583 | 1.00 (Reference) | 1.40 (1.03, 1.90) | 1.12 (0.91, 1.37) | 1.24 (0.86, 1.81) | 1.18 (0.93, 1.49) | 0.047 | 0.625 |
| Past smoker | 2447/10476 | 1.00 (Reference) | 1.28 (0.93, 1.74) | 1.02 (0.84, 1.24) | 1.06 (0.69, 1.64) | 1.12 (0.90, 1.38) | 0.295 | 0.625 |
| Current smoker | 1229/8685 | 1.00 (Reference) | 1.22 (0.79, 1.86) | 0.93 (0.68, 1.28) | 0.70 (0.41, 1.20) | 1.22 (0.95, 1.56) | 0.395 | 0.625 |
| **Drinking status** |  |  |  |  |  |  |  |  |
| Never drinker | 1053/6015 | 1.00 (Reference) | 1.16 (0.85, 1.58) | 1.00 (0.62, 1.62) | 1.50 (0.91, 2.50) | 1.14 (0.80, 1.63) | 0.176 | 0.85 |
| Past drinker | 2085/7439 | 1.00 (Reference) | 0.95 (0.76, 1.18) | 0.82 (0.55, 1.23) | 1.23 (0.88, 1.72) | 1.31 (1.08, 1.59) | 0.017 | 0.85 |
| Current drinker | 2928/28290 | 1.00 (Reference) | 1.06 (0.90, 1.24) | 1.26 (0.91, 1.76) | 1.29 (0.98, 1.70) | 1.19 (1.00, 1.42) | 0.003 | 0.85 |
| **HEI-2015 score** |  |  |  |  |  |  |  |  |
| <70 | 586/3743 | 1.00 (Reference) | 1.16 (0.79, 1.68) | 0.53 (0.17, 1.67) | 1.84 (1.02, 3.32) | 1.18 (0.65, 2.13) | 0.147 | 0.795 |
| ≥70 | 5480/38001 | 1.00 (Reference) | 1.01 (0.88, 1.15) | 1.10 (0.87, 1.39) | 1.22 (0.98, 1.51) | 1.23 (1.08, 1.42) | 0.001 | 0.795 |
| **Sleep hours** |  |  |  |  |  |  |  |  |
| <6 hours | 901/6189 | 1.00 (Reference) | 1.37 (0.87, 2.15) | 1.03 (0.77, 1.37) | 1.05 (0.70, 1.56) | 1.22 (0.93, 1.61) | 0.153 | 0.757 |
| ≥6 hours | 5165/35555 | 1.00 (Reference) | 1.28 (1.02, 1.61) | 1.03 (0.90, 1.18) | 1.02 (0.77, 1.35) | 1.20 (1.03, 1.39) | 0.03 | 0.757 |
|  |  |  |  |  |  |  |  |  |
| Cancer mortality |  |  |  |  |  |  |  |  |
| **Age (years)** |  |  |  |  |  |  |  |  |
| age<65 | 502/31181 | 1.00 (Reference) | 0.86 (0.56, 1.32) | 0.92 (0.49, 1.72) | 1.21 (0.76, 1.94) | 1.05 (0.71, 1.56) | 0.687 | 0.621 |
| age≥65 | 879/10563 | 1.00 (Reference) | 0.80 (0.57, 1.11) | 1.27 (0.66, 2.46) | 0.93 (0.52, 1.67) | 1.21 (0.91, 1.60) | 0.486 | 0.621 |
| **Sex** |  |  |  |  |  |  |  |  |
| Men | 829/20602 | 1.00 (Reference) | 0.92 (0.65, 1.30) | 1.31 (0.67, 2.57) | 1.25 (0.77, 2.04) | 1.36 (0.94, 1.97) | 0.078 | 0.958 |
| Women | 552/21142 | 1.00 (Reference) | 0.75 (0.52, 1.08) | 1.07 (0.63, 1.83) | 1.20 (0.68, 2.13) | 1.26 (0.78, 2.06) | 0.401 | 0.958 |
| **Body mass index (kg/m^2^)** |  |  |  |  |  |  |  |  |
| >25 | 397/12161 | 1.00 (Reference) | 0.78 (0.45, 1.34) | 1.24 (0.63, 2.45) | 1.06 (0.53, 2.14) | 1.75 (1.15, 2.67) | 0.029 | 0.301 |
| 25-29 | 495/14064 | 1.00 (Reference) | 0.82 (0.55, 1.22) | 1.03 (0.40, 2.69) | 1.69 (1.02, 2.82) | 1.03 (0.64, 1.65) | 0.469 | 0.301 |
| ≥30 | 489/15519 | 1.00 (Reference) | 0.88 (0.55, 1.39) | 1.31 (0.72, 2.39) | 0.91 (0.42, 1.94) | 1.25 (0.74, 2.10) | 0.488 | 0.301 |
| **Smoking status** |  |  |  |  |  |  |  |  |
| Never smoker | 456/22583 | 1.00 (Reference) | 1.64 (0.87, 3.11) | 0.97 (0.58, 1.62) | 1.72 (0.72, 4.08) | 1.37 (0.79, 2.37) | 0.171 | 0.818 |
| Past smoker | 581/10476 | 1.00 (Reference) | 1.08 (0.55, 2.11) | 0.83 (0.54, 1.27) | 1.07 (0.51, 2.27) | 0.69 (0.45, 1.04) | 0.127 | 0.818 |
| Current smoker | 344/8685 | 1.00 (Reference) | 1.14 (0.74, 1.76) | 0.73 (0.42, 1.27) | 0.74 (0.39, 1.38) | 1.70 (1.08, 2.65) | 0.111 | 0.818 |
| **Drinking status** |  |  |  |  |  |  |  |  |
| Never drinker | 181/6015 | 1.00 (Reference) | 1.71 (0.86, 3.40) | 2.19 (0.85, 5.62) | 3.02 (1.09, 8.41) | 1.01 (0.43, 2.39) | 0.141 | 0.185 |
| Past drinker | 477/7439 | 1.00 (Reference) | 0.76 (0.45, 1.27) | 0.87 (0.38, 1.99) | 1.42 (0.74, 2.75) | 1.30 (0.82, 2.04) | 0.295 | 0.185 |
| Current drinker | 723/28290 | 1.00 (Reference) | 0.75 (0.54, 1.04) | 1.20 (0.67, 2.14) | 0.98 (0.62, 1.54) | 1.40 (0.96, 2.03) | 0.166 | 0.185 |
| **HEI-2015 score** |  |  |  |  |  |  |  |  |
| <70 | 129/3743 | 1.00 (Reference) | 1.08 (0.46, 2.52) | 0.81 (0.10, 6.62) | 1.94 (0.69, 5.43) | 1.22 (0.36, 4.17) | 0.408 | 0.874 |
| ≥70 | 1252/38001 | 1.00 (Reference) | 0.81 (0.61, 1.08) | 1.25 (0.79, 1.97) | 1.15 (0.81, 1.64) | 1.34 (1.01, 1.78) | 0.047 | 0.874 |
| **Sleep hours** |  |  |  |  |  |  |  |  |
| <6 hours | 211/6189 | 1.00 (Reference) | 2.40 (1.10, 5.27) | 0.89 (0.50, 1.58) | 1.23 (0.53, 2.84) | 1.64 (0.91, 2.98) | 0.155 | 0.41 |
| ≥6 hours | 1170/35555 | 1.00 (Reference) | 1.00 (0.64, 1.56) | 0.82 (0.61, 1.11) | 1.13 (0.68, 1.88) | 1.21 (0.89, 1.63) | 0.486 | 0.41 |
|  |  |  |  |  |  |  |  |  |
| Diabetes mortality |  |  |  |  |  |  |  |  |
| **Age (years)** |  |  |  |  |  |  |  |  |
| age<65 | 87/31181 | 1.00 (Reference) | 0.72 (0.24, 2.18) | 2.25 (0.52, 9.75) | 2.51 (0.81, 7.81) | 1.82 (0.89, 3.72) | 0.031 | 0.85 |
| age≥65 | 119/10563 | 1.00 (Reference) | 2.04 (1.07, 3.92) | 1.39 (0.44, 4.42) | 0.35 (0.06, 2.18) | 1.97 (0.68, 5.70) | 0.19 | 0.85 |
| **Sex** |  |  |  |  |  |  |  |  |
| Men | 104/20602 | 1.00 (Reference) | 0.66 (0.21, 2.03) | 3.11 (0.98, 9.82) | 3.18 (1.00, 10.19) | 2.46 (1.09, 5.52) | 0.005 | 0.139 |
| Women | 102/21142 | 1.00 (Reference) | 1.75 (0.84, 3.65) | 0.73 (0.22, 2.43) | 0.25 (0.04, 1.60) | 1.46 (0.52, 4.11) | 0.616 | 0.139 |
| **Body mass index (kg/m^2^)** |  |  |  |  |  |  |  |  |
| >25 | 45/12161 | 1.00 (Reference) | 0.43 (0.08, 2.42) | 2.33 (0.96, 5.69) | 0.17 (0.02, 1.27) | 0.00 (0.00, 0.00) | 0.106 | 0.594 |
| 25-29 | 62/14064 | 1.00 (Reference) | 1.95 (0.74, 5.16) | 1.15 (0.31, 4.23) | 3.16 (0.50, 20.12) | 4.12 (1.71, 9.94) | 0.001 | 0.594 |
| ≥30 | 99/15519 | 1.00 (Reference) | 1.46 (0.68, 3.16) | 0.45 (0.07, 2.77) | 2.34 (0.62, 8.74) | 1.69 (0.78, 3.63) | 0.104 | 0.594 |
| **Smoking status** |  |  |  |  |  |  |  |  |
| Never smoker | 99/22583 | 1.00 (Reference) | 2.10 (0.46, 9.66) | 1.89 (0.95, 3.76) | 0.67 (0.11, 4.27) | 1.43 (0.55, 3.74) | 0.208 | 0.947 |
| Past smoker | 75/10476 | 1.00 (Reference) | 2.97 (0.66, 13.42) | 0.84 (0.25, 2.80) | 1.03 (0.21, 5.01) | 2.84 (1.23, 6.58) | 0.035 | 0.947 |
| Current smoker | 32/8685 | 1.00 (Reference) | 0.00 (0.00, 0.00) | 0.33 (0.05, 2.05) | 2.83 (1.45, 5.52) | 0.90 (0.23, 3.46) | 0.905 | 0.947 |
| **Drinking status** |  |  |  |  |  |  |  |  |
| Never drinker | 63/6015 | 1.00 (Reference) | 1.66 (0.66, 4.21) | 2.67 (0.68, 10.54) | 1.12 (0.30, 4.18) | 1.67 (0.55, 5.06) | 0.228 | 0.368 |
| Past drinker | 69/7439 | 1.00 (Reference) | 1.06 (0.28, 3.95) | 0.12 (0.01, 1.05) | 1.71 (0.31, 9.42) | 3.84 (1.77, 8.31) | 0.003 | 0.368 |
| Current drinker | 74/28290 | 1.00 (Reference) | 1.29 (0.56, 3.00) | 3.61 (0.94, 13.85) | 2.55 (0.60, 10.92) | 0.66 (0.19, 2.30) | 0.605 | 0.368 |
| **HEI-2015 score** |  |  |  |  |  |  |  |  |
| <70 | 21/3743 | 1.00 (Reference) | 0.25 (0.03, 2.06) | 0.00 (0.00, 0.00) | 0.00 (0.00, 0.00) | 2.24 (0.63, 7.96) | 0.911 | 0.931 |
| ≥70 | 185/38001 | 1.00 (Reference) | 1.31 (0.74, 2.30) | 1.88 (0.54, 6.55) | 2.40 (0.85, 6.76) | 1.90 (1.02, 3.56) | 0.008 | 0.931 |
| **Sleep hours** |  |  |  |  |  |  |  |  |
| <6 hours | 40/6189 | 1.00 (Reference) | 0.08 (0.01, 0.81) | 0.47 (0.06, 3.57) | 1.98 (0.44, 8.82) | 1.37 (0.60, 3.12) | 0.485 | 0.48 |
| ≥6 hours | 166/35555 | 1.00 (Reference) | 2.96 (1.01, 8.64) | 1.44 (0.78, 2.65) | 1.89 (0.44, 8.21) | 2.19 (1.11, 4.32) | 0.009 | 0.48 |

Abbreviations: aHR, adjusted hazard ratio; CI, confidence intervals; Ref., reference; HEI-2015, Healthy Eating Index 2015; CVD, cardiovascular disease.

* aHR (95%CI) was estimated by weighted Cox regression analyses. Date is shown as aHR with 95%CI.

Model adjusted for age, sex, education, race/ethnicity, family income, BMI, dietary energy intake, drinking status, smoking status, physical activity, diabetes, hypertension, hyperlipidemia, CVD, cancer, adherence to HEI-2015 score, and dietary supplement use, except for subgroup variables..

# Supplementary Table 10 Association between timing of night eating (21:00 to 4:00) with all-cause, cancer, and diabetes mortality

| **Cause of mortality** | **Timing of night eating** | | | | | | | | ***P* trend** |
| --- | --- | --- | --- | --- | --- | --- | --- | --- | --- |
|  | **No night eating** | **21:00~22:00** | **22:00~23:00** | **23:00~00:00** | **00:00~1:00** | **1:00~2:00** | **2:00~3:00** | **3:00~4:00** |  |
| All-cause mortality |  |  |  |  |  |  |  |  |  |
| Case/N | 3898/23581 | 1012/7781 | 741/6262 | 244/2354 | 46/588 | 39/417 | 45/375 | 41/386 |  |
| Model 1 | 1 (Ref.) | 0.93 (0.85, 1.02) | 1.05 (0.93, 1.18) | 1.36 (1.14, 1.62) | 1.42 (1.02, 1.96) | 1.58 (1.00, 2.47) | 1.28 (0.87, 1.90) | 1.32 (0.87, 2.00) | <0.001 |
| Model 2 | 1 (Ref.) | 0.96 (0.88, 1.05) | 1.08 (0.97, 1.21) | 1.32 (1.10, 1.58) | 1.42 (1.03, 1.96) | 1.53 (0.99, 2.38) | 1.22 (0.81, 1.83) | 1.27 (0.84, 1.92) | <0.001 |
| Model 3 | 1 (Ref.) | 0.93 (0.85, 1.02) | 1.03 (0.92, 1.14) | 1.24 (1.04, 1.49) | 1.40 (1.03, 1.92) | 1.44 (0.94, 2.21) | 1.19 (0.79, 1.78) | 1.21 (0.79, 1.85) | 0.007 |
| Cancer mortality |  |  |  |  |  |  |  |  |  |
| Case/N | 889/23581 | 214/7781 | 179/6262 | 52/2354 | 14/588 | 14/417 | 9/375 | 10/386 |  |
| Model 1 | 1 (Ref.) | 0.89 (0.72, 1.10) | 1.04 (0.85, 1.27) | 1.00 (0.64, 1.58) | 1.47 (0.79, 2.72) | 2.02 (0.97, 4.21) | 1.06 (0.47, 2.37) | 1.02 (0.40, 2.58) | 0.357 |
| Model 2 | 1 (Ref.) | 0.91 (0.74, 1.13) | 1.05 (0.86, 1.28) | 0.98 (0.62, 1.55) | 1.47 (0.81, 2.67) | 1.99 (0.96, 4.13) | 1.02 (0.45, 2.30) | 1.00 (0.40, 2.51) | 0.369 |
| Model 3 | 1 (Ref.) | 0.89 (0.72, 1.11) | 1.03 (0.84, 1.26) | 0.93 (0.59, 1.47) | 1.53 (0.83, 2.82) | 2.01 (1.00, 4.05) | 0.94 (0.42, 2.10) | 0.87 (0.35, 2.17) | 0.607 |
| Diabetes mortality |  |  |  |  |  |  |  |  |  |
| Case/N | 139/23581 | 23/7781 | 27/6262 | 14/2354 | 1/588 | 1/417 | 1/375 | 0/386 |  |
| Model 1 | 1 (Ref.) | 0.36 (0.20, 0.67) | 1.66 (0.99, 2.79) | 2.27 (1.16, 4.45) | 0.39 (0.06, 2.82) | 0.92 (0.12, 7.29) | 0.41 (0.05, 3.06) | 0.00 (0.00, 0.00) | 0.323 |
| Model 2 | 1 (Ref.) | 0.40 (0.21, 0.74) | 1.82 (1.10, 3.02) | 2.33 (1.18, 4.57) | 0.46 (0.06, 3.42) | 1.03 (0.13, 8.19) | 0.45 (0.06, 3.29) | 0.00 (0.00, 0.00) | 0.172 |
| Model 3 | 1 (Ref.) | 0.36 (0.19, 0.68) | 1.47 (0.92, 2.36) | 1.90 (0.98, 3.66) | 0.53 (0.07, 4.12) | 0.90 (0.12, 7.07) | 0.55 (0.07, 4.12) | 0.00 (0.00, 0.00) | 0.372 |

Abbreviations: aHR, adjusted hazard ratio; CI, confidence intervals; Ref., reference; HEI-2015, Healthy Eating Index 2015; CVD, cardiovascular disease.

* aHR (95%CI) was estimated by weighted Cox regression analyses. Date is shown as aHR with 95%CI.

Model 1 adjusted for, age and sex.

Model 2 further adjusted for education, race/ethnicity, family income, and body mass index.

Model 3 further adjusted for dietary energy intake, drinking status, smoking status, physical activity, diabetes, hypertension,hyperlipidemia, CVD, cancer, adherence to HEI-2015 score, and dietary supplement use.

# Supplementary Table 11 Association between night eating frequency (21:00 to 4:00) with all-cause, cancer, and diabetes mortality

| **Cause of mortality** | **Night eating frequency** | | | ***P* trend** |
| --- | --- | --- | --- | --- |
|  | **No night eating** | **One time** | **Two times or over** |  |
| All-cause mortality |  |  |  |  |
| Case/N | 3898/23581 | 1913/15339 | 255/2824 |  |
| Model 1 | 1.00 (Reference) | 1.03 (0.96, 1.10) | 1.15 (0.95, 1.38) | 0.151 |
| Model 2 | 1.00 (Reference) | 1.05 (0.98, 1.12) | 1.20 (0.99, 1.44) | 0.041 |
| Model 3 | 1.00 (Reference) | 1.01 (0.94, 1.08) | 1.15 (0.95, 1.38) | 0.301 |
| Cancer mortality |  |  |  |  |
| Case/N | 889/23581 | 423/15339 | 69/2824 |  |
| Model 1 | 1.00 (Reference) | 0.96 (0.84, 1.11) | 1.13 (0.81, 1.56) | 0.916 |
| Model 2 | 1.00 (Reference) | 0.98 (0.84, 1.13) | 1.15 (0.83, 1.59) | 0.788 |
| Model 3 | 1.00 (Reference) | 0.95 (0.82, 1.10) | 1.12 (0.81, 1.55) | 0.982 |
| Diabetes mortality |  |  |  |  |
| Case/N | 139/23581 | 60/15339 | 7/2824 |  |
| Model 1 | 1.00 (Reference) | 0.99 (0.66, 1.48) | 1.04 (0.42, 2.56) | 0.992 |
| Model 2 | 1.00 (Reference) | 1.06 (0.71, 1.59) | 1.19 (0.48, 2.91) | 0.66 |
| Model 3 | 1.00 (Reference) | 0.93 (0.63, 1.37) | 1.06 (0.43, 2.60) | 0.851 |

Abbreviations: aHR, adjusted hazard ratio; CI, confidence intervals; Ref., reference; HEI-2015, Healthy Eating Index 2015; CVD, cardiovascular disease.

* aHR (95%CI) was estimated by weighted Cox regression analyses. Date is shown as aHR with 95%CI.

Model 1 adjusted for, age and sex.

Model 2 further adjusted for education, race/ethnicity, family income, and body mass index.

Model 3 further adjusted for dietary energy intake, drinking status, smoking status, physical activity, diabetes, hypertension, hyperlipidemia, CVD, cancer, adherence to HEI-2015 score, and dietary supplement use.

# Supplementary Table 12 Association between food quality of night eating (21:00 to 4:00) with all-cause, cancer, and diabetes mortality

| **Cause of mortality** | **Food quality of night eating** | | | | | ***P* trend** |
| --- | --- | --- | --- | --- | --- | --- |
|  | **No night eating** | **VL-energy intake** | **L-energy intake** | **M-energy intake** | **H-energy intake** |  |
| All-cause mortality |  |  |  |  |  |  |
| Case/N | 3898/23581 | 318/2344 | 318/2634 | 371/3723 | 1161/9462 |  |
| Model 1 | 1.00 (Reference) | 1.09 (0.94, 1.27) | 0.99 (0.84, 1.16) | 1.37 (1.16, 1.62) | 0.96 (0.88, 1.05) | 0.032 |
| Model 2 | 1.00 (Reference) | 1.15 (0.99, 1.33) | 1.04 (0.90, 1.21) | 1.32 (1.12, 1.55) | 0.98 (0.90, 1.07) | 0.006 |
| Model 3 | 1.00 (Reference) | 1.06 (0.92, 1.23) | 1.00 (0.86, 1.17) | 1.24 (1.05, 1.46) | 0.95 (0.87, 1.03) | 0.097 |
| Cancer mortality |  |  |  |  |  |  |
| Case/N | 889/23581 | 65/2344 | 69/2634 | 105/3723 | 253/9462 |  |
| Model 1 | 1.00 (Reference) | 1.00 (0.73, 1.37) | 0.81 (0.60, 1.11) | 1.39 (1.05, 1.85) | 0.92 (0.76, 1.11) | 0.808 |
| Model 2 | 1.00 (Reference) | 1.02 (0.75, 1.40) | 0.84 (0.62, 1.14) | 1.35 (1.01, 1.80) | 0.93 (0.77, 1.13) | 0.717 |
| Model 3 | 1.00 (Reference) | 0.98 (0.72, 1.33) | 0.82 (0.60, 1.12) | 1.30 (0.99, 1.71) | 0.91 (0.75, 1.10) | 0.973 |
| Diabetes mortality |  |  |  |  |  |  |
| Case/N | 139/23581 | 9/2344 | 16/2634 | 10/3723 | 32/9462 |  |
| Model 1 | 1.00 (Reference) | 1.05 (0.35, 3.13) | 1.93 (0.99, 3.77) | 1.20 (0.53, 2.71) | 0.66 (0.38, 1.16) | 0.148 |
| Model 2 | 1.00 (Reference) | 1.24 (0.42, 3.70) | 2.17 (1.13, 4.16) | 1.23 (0.55, 2.74) | 0.71 (0.41, 1.25) | 0.067 |
| Model 3 | 1.00 (Reference) | 0.95 (0.35, 2.62) | 1.71 (0.92, 3.17) | 1.05 (0.46, 2.39) | 0.65 (0.37, 1.14) | 0.261 |

Abbreviations: aHR, adjusted hazard ratio; CI, confidence intervals; Ref., reference; HEI-2015, Healthy Eating Index 2015; CVD, cardiovascular disease.

* aHR (95%CI) was estimated by weighted Cox regression analyses. Date is shown as aHR with 95%CI.

Model 1 adjusted for, age and sex.

Model 2 further adjusted for education, race/ethnicity, family income, and body mass index.

Model 3 further adjusted for dietary energy intake, drinking status, smoking status, physical activity, diabetes, hypertension, hyperlipidemia, CVD, cancer, adherence to HEI-2015 score, and dietary supplement use.

**Supplementary Table 13 Association between timing of night eating with all-cause, cancer, and diabetes mortality after excluding participants whose nocturnal energy intake exceeds 50%**

| **Cause of mortality** | **Timing of night eating** | | | | | | | ***P* trend** |
| --- | --- | --- | --- | --- | --- | --- | --- | --- |
|  | **No night eating** | **22:00~23:00** | **23:00~00:00** | **00:00~1:00** | **1:00~2:00** | **2:00~3:00** | **3:00~4:00** |  |
| All-cause mortality |  |  |  |  |  |  |  |  |
| Case/N | 4910/31362 | 718/5944 | 229/2129 | 39/489 | 38/362 | 44/314 | 40/334 |  |
| Model 1 | 1.00 (Reference) | 1.05 (0.94, 1.18) | 1.36 (1.14, 1.63) | 1.35 (0.92, 1.99) | 1.65 (1.06, 2.59) | 1.32 (0.88, 1.99) | 1.39 (0.91, 2.12) | <0.001 |
| Model 2 | 1.00 (Reference) | 1.10 (0.98, 1.22) | 1.33 (1.11, 1.60) | 1.32 (0.89, 1.96) | 1.61 (1.04, 2.50) | 1.25 (0.82, 1.91) | 1.31 (0.86, 2.00) | <0.001 |
| Model 3 | 1.00 (Reference) | 1.03 (0.93, 1.15) | 1.29 (1.08, 1.55) | 1.31 (0.88, 1.94) | 1.54 (1.00, 2.36) | 1.14 (0.73, 1.76) | 1.26 (0.82, 1.93) | 0.003 |
| Cancer mortality |  |  |  |  |  |  |  |  |
| Case/N | 1103/31362 | 171/5944 | 47/2129 | 12/489 | 14/362 | 9/314 | 10/334 |  |
| Model 1 | 1.00 (Reference) | 1.02 (0.82, 1.26) | 0.91 (0.59, 1.40) | 1.52 (0.76, 3.00) | 2.27 (1.08, 4.76) | 1.18 (0.53, 2.63) | 1.11 (0.44, 2.83) | 0.281 |
| Model 2 | 1.00 (Reference) | 1.03 (0.84, 1.27) | 0.89 (0.58, 1.37) | 1.49 (0.76, 2.92) | 2.23 (1.07, 4.68) | 1.13 (0.50, 2.54) | 1.07 (0.42, 2.70) | 0.312 |
| Model 3 | 1.00 (Reference) | 1.01 (0.82, 1.25) | 0.85 (0.55, 1.32) | 1.60 (0.81, 3.14) | 2.29 (1.12, 4.68) | 1.01 (0.46, 2.22) | 0.93 (0.37, 2.35) | 0.524 |
| Diabetes mortality |  |  |  |  |  |  |  |  |
| Case/N | 162/31362 | 25/5944 | 14/2129 | 1/489 | 1/362 | 1/314 | 0/334 |  |
| Model 1 | 1.00 (Reference) | 1.95 (1.16, 3.27) | 2.87 (1.46, 5.62) | 0.54 (0.08, 3.87) | 1.20 (0.15, 9.55) | 0.53 (0.07, 3.90) | 0.00 (0.00, 0.00) | 0.009 |
| Model 2 | 1.00 (Reference) | 2.13 (1.29, 3.51) | 2.84 (1.42, 5.66) | 0.60 (0.08, 4.44) | 1.31 (0.16, 10.46) | 0.58 (0.08, 4.21) | 0.00 (0.00, 0.00) | 0.003 |
| Model 3 | 1.00 (Reference) | 1.67 (1.05, 2.65) | 2.50 (1.30, 4.82) | 0.65 (0.08, 5.05) | 1.23 (0.16, 9.57) | 0.63 (0.08, 4.69) | 0.00 (0.00, 0.00) | 0.017 |

Abbreviations: aHR, adjusted hazard ratio; CI, confidence intervals; Ref., reference; HEI-2015, Healthy Eating Index 2015; CVD, cardiovascular disease.

* aHR (95%CI) was estimated by weighted Cox regression analyses. Date is shown as aHR with 95%CI.

Model 1 adjusted for, age and sex.

Model 2 further adjusted for education, race/ethnicity, family income, and body mass index.

Model 3 further adjusted for dietary energy intake, drinking status, smoking status, physical activity, diabetes, hypertension, hyperlipidemia, CVD, cancer, adherence to HEI-2015 score, and dietary supplement use.

**Supplementary Table 14 Association between night eating frequency with all-cause, cancer, and diabetes mortality after excluding participants whose nocturnal energy intake exceeds 50%**

| **Cause of mortality** | **Night eating frequency** | | | ***P* trend** |
| --- | --- | --- | --- | --- |
|  | **No night eating** | **One time** | **Two times or over** |  |
| All-cause mortality |  |  |  |  |
| Case/N | 4910/31362 | 997/8543 | 111/1029 |  |
| Model 1 | 1.00 (Reference) | 1.12 (1.02, 1.23) | 1.54 (1.19, 1.99) | <0.001 |
| Model 2 | 1.00 (Reference) | 1.14 (1.04, 1.24) | 1.59 (1.25, 2.04) | <0.001 |
| Model 3 | 1.00 (Reference) | 1.08 (0.99, 1.18) | 1.49 (1.16, 1.90) | 0.002 |
| Cancer mortality |  |  |  |  |
| Case/N | 1103/31362 | 234/8543 | 29/1029 |  |
| Model 1 | 1.00 (Reference) | 1.04 (0.86, 1.25) | 1.27 (0.74, 2.20) | 0.423 |
| Model 2 | 1.00 (Reference) | 1.05 (0.87, 1.26) | 1.31 (0.76, 2.25) | 0.346 |
| Model 3 | 1.00 (Reference) | 1.01 (0.84, 1.22) | 1.21 (0.71, 2.08) | 0.609 |
| Diabetes mortality |  |  |  |  |
| Case/N | 162/31362 | 40/8543 | 2/1029 |  |
| Model 1 | 1.00 (Reference) | 1.99 (1.30, 3.05) | 1.13 (0.27, 4.78) | 0.004 |
| Model 2 | 1.00 (Reference) | 2.12 (1.41, 3.19) | 1.29 (0.30, 5.50) | 0.001 |
| Model 3 | 1.00 (Reference) | 1.74 (1.20, 2.54) | 1.25 (0.29, 5.44) | 0.013 |

Abbreviations: aHR, adjusted hazard ratio; CI, confidence intervals; Ref., reference; HEI-2015, Healthy Eating Index 2015; CVD, cardiovascular disease.

* aHR (95%CI) was estimated by weighted Cox regression analyses. Date is shown as aHR with 95%CI.

Model 1 adjusted for, age and sex.

Model 2 further adjusted for education, race/ethnicity, family income, and body mass index.

Model 3 further adjusted for dietary energy intake, drinking status, smoking status, physical activity, diabetes, hypertension, hyperlipidemia, CVD, cancer, adherence to HEI-2015 score, and dietary supplement use.

**Supplementary Table 15 Association between food quality of night eating with all-cause, cancer, and diabetes mortality after excluding participants whose nocturnal energy intake exceeds 50%**

| **Cause of mortality** | **Food quality** | | | | | ***P* trend** |
| --- | --- | --- | --- | --- | --- | --- |
|  | **No night eating** | **VL-energy intake** | **L-energy intake** | **M-energy intake** | **H-energy intake** |  |
| All-cause mortality |  |  |  |  |  |  |
| Case/N | 4910/31362 | 177/1772 | 436/3113 | 140/1205 | 355/3482 |  |
| Model 1 | 1.00 (Reference) | 1.25 (1.01, 1.54) | 1.03 (0.91, 1.16) | 1.13 (0.87, 1.45) | 1.31 (1.13, 1.51) | 0.001 |
| Model 2 | 1.00 (Reference) | 1.30 (1.05, 1.60) | 1.09 (0.97, 1.23) | 1.11 (0.87, 1.42) | 1.25 (1.08, 1.45) | 0.001 |
| Model 3 | 1.00 (Reference) | 1.28 (1.03, 1.58) | 1.04 (0.92, 1.18) | 1.02 (0.80, 1.31) | 1.17 (1.01, 1.35) | 0.034 |
| Cancer mortality |  |  |  |  |  |  |
| Case/N | 1103/31362 | 46/1772 | 89/3113 | 34/1205 | 94/3482 |  |
| Model 1 | 1.00 (Reference) | 1.17 (0.83, 1.66) | 0.84 (0.65, 1.08) | 1.21 (0.76, 1.92) | 1.25 (0.93, 1.68) | 0.306 |
| Model 2 | 1.00 (Reference) | 1.19 (0.84, 1.68) | 0.87 (0.68, 1.12) | 1.18 (0.75, 1.86) | 1.20 (0.90, 1.61) | 0.37 |
| Model 3 | 1.00 (Reference) | 1.21 (0.86, 1.71) | 0.84 (0.65, 1.08) | 1.14 (0.72, 1.81) | 1.15 (0.86, 1.55) | 0.594 |
| Diabetes mortality |  |  |  |  |  |  |
| Case/N | 162/31362 | 5/1772 | 17/3113 | 6/1205 | 14/3482 |  |
| Model 1 | 1.00 (Reference) | 2.04 (0.67, 6.20) | 1.37 (0.76, 2.49) | 2.15 (0.55, 8.44) | 2.50 (1.33, 4.71) | 0.002 |
| Model 2 | 1.00 (Reference) | 2.37 (0.80, 7.06) | 1.56 (0.87, 2.82) | 2.20 (0.57, 8.45) | 2.42 (1.29, 4.52) | 0.001 |
| Model 3 | 1.00 (Reference) | 2.11 (0.73, 6.14) | 1.29 (0.73, 2.26) | 1.86 (0.55, 6.26) | 2.04 (1.14, 3.66) | 0.006 |

Abbreviations: aHR, adjusted hazard ratio; CI, confidence intervals; Ref., reference; HEI-2015, Healthy Eating Index 2015; CVD, cardiovascular disease.

* aHR (95%CI) was estimated by weighted Cox regression analyses. Date is shown as aHR with 95%CI.

Model 1 adjusted for, age and sex.

Model 2 further adjusted for education, race/ethnicity, family income, and body mass index.

Model 3 further adjusted for dietary energy intake, drinking status, smoking status, physical activity, diabetes, hypertension, hyperlipidemia, CVD, cancer, adherence to HEI-2015 score, and dietary supplement use.
